# Plant-Derived Food Grade Substances (PDFGS) Active Against Respiratory Viruses: A Systematic Review of Non-clinical Studies

**DOI:** 10.3389/fnut.2021.606782

**Published:** 2021-02-09

**Authors:** Francis U. Umeoguaju, Benson C. Ephraim-Emmanuel, Kingsley C. Patrick-Iwuanyanwu, Judith T. Zelikoff, Orish Ebere Orisakwe

**Affiliations:** ^1^World Bank Africa Centre of Excellence in Public Health and Toxicological Research (ACE-PUTOR), University of Port Harcourt, Port Harcourt, Nigeria; ^2^Department of Dental Health Sciences, Ogbia, Bayelsa State College of Health Technology, Otakeme, Nigeria; ^3^Department of Environmental Medicine, New York University Grossman School of Medicine, New York, NY, United States; ^4^Department of Experimental Pharmacology and Toxicology, Faculty of Pharmacy, University of Port Harcourt, Port Harcourt, Nigeria

**Keywords:** IAV, RSV, HCoV, Antiviral agent, Respiratory Tract Infection (RTI), functional foods, Viral lifecyle, polyphenols

## Abstract

Human diet comprises several classes of phytochemicals some of which are potentially active against human pathogenic viruses. This study examined available evidence that identifies existing food plants or constituents of edible foods that have been reported to inhibit viral pathogenesis of the human respiratory tract. SCOPUS and PUBMED databases were searched with keywords designed to retrieve articles that investigated the effect of plant-derived food grade substances (PDFGS) on the activities of human pathogenic viruses. Eligible studies for this review were those done on viruses that infect the human respiratory tract. Forty six (46) studies met the specified inclusion criteria from the initial 5,734 hits. The selected studies investigated the effects of different PDFGS on the infectivity, proliferation and cytotoxicity of different respiratory viruses including influenza A virus (IAV), influenza B virus (IBV), Respiratory syncytial virus (RSV), human parainfluenza virus (hPIV), Human coronavirus NL63 (HCoV-NL63), and rhinovirus (RV) in cell lines and mouse models. This review reveals that PDFGS inhibits different stages of the pathological pathways of respiratory viruses including cell entry, replication, viral release and viral-induced dysregulation of cellular homeostasis and functions. These alterations eventually lead to the reduction of virus titer, viral-induced cellular damages and improved survival of host cells. Major food constituents active against respiratory viruses include flavonoids, phenolic acids, tannins, lectins, vitamin D, curcumin, and plant glycosides such as glycyrrhizin, acteoside, geniposide, and iridoid glycosides. Herbal teas such as guava tea, green and black tea, adlay tea, cistanche tea, kuding tea, licorice extracts, and edible bird nest extracts were also effective against respiratory viruses *in vitro*. The authors of this review recommend an increased consumption of foods rich in these PDFGS including legumes, fruits (e.g berries, citrus), tea, fatty fish and curcumin amongst human populations with high prevalence of respiratory viral infections in order to prevent, manage and/or reduce the severity of respiratory virus infections.

## Introduction

Several people suffer yearly from respiratory tract infections caused by viruses and a host of other organisms (1). Infections of the respiratory tracts are leading cause of death in children below 5 years with up to 500,000 annual deaths globally (2). Respiratory viruses are major contributor to respiratory tract infections. Influenza viruses alone is reported to affect about 20% of the world's population resulting in an annual mortality of over 500,000 globally (3, 4). Human respiratory syncytial virus (RSV) is also reported to cause over 30 million cases of lower respiratory tract infection amongst children every year. This consequently leads to over 3 million hospitalization and about 60,000 deaths amongst infected persons (5). Over 200 strains of Respiratory viruses belonging to the families of adenoviridae, parvoviridae, orthomyxoviridae, paramyxoviridae, picornaviridae, and coronaviridae have been reported (2). Some notable examples of pathogenic viruses known to infect human respiratory tract includes influenza viruses, human parainfluenza virus (hPIV), RSV, adenovirus (AdV), rhinovirus (RV), enterovirus, parechovirus, human metapneumovirus, coronavirus, human bocavirus, parvovirus (type 4 and 5), and mimivirus (2, 6, 7).

Respiratory viruses are highly infective and are transmitted through contact with virus containing substances such as respiratory secretions, stools and urine (6). These viruses readily infect the upper respiratory tracts and cause mild infections including cold and flu. However, symptoms aggravates once infections gets to the lower respiratory tracts (2, 8). Most respiratory viruses have seasonal outbreaks while some like hPIV infection occurs all year round (7). Some of the general symptoms of respiratory viruses include seasonal colds, bronchiolitis (especially with RSV), acute otitis, sinisitis, croup (mainly by hPIV), worsening of chronic obstructive pulmonary disease (COPD) and asthma, pneumonia (7). The most vulnerable groups to respiratory virus infection include immunocompromised patients, elderly, and infants (2, 7, 9).

Some of these viruses have been reported to cause pathology by inducing severe oxidative stress and significantly reducing the expressions of nuclear factor erythroid 2-related factor 2 (Nrf2) and heme oxygenase-1 as well as cause the activation of toll-like receptor (TLR) signaling pathways amongst other pathological mechanisms (10, 11). This notwithstanding, in the event of occurrence of a viral infection, the body's immune system stages innate and adaptive immune responses that recognize and destroy the viral threat as well as resolve inflammation and repair the damages caused by these viruses in the body (12).

Over the years in different continents of the world, natural plant products which are edible and known to possess medicinal properties have been continually used for the treatment of infections and disease ailments (13). Research on these plant-derived food grade substances (PDFGS) has come at a time when there are increasing cases of resistance to conventional antimicrobials as well as issues with potency, safety amongst others (13). These PDFGS provide a wide variety of treatment options that can be applied in modern medicine as either a supplementary or a main treatment modality. In actual fact, up to 80% of populations resident in developing countries apply these natural remedies in one way or the other for the treatment of diseases, supplementation of body nutrition, boosting the immune system amongst other applications (13–15). Some PDFGS such as adlay tea, *Houttuynia cordata (H. cordata)* Thunb are widely used in traditional medicine in the management of different respiratory tract infections (16, 17).

It is also necessary to point out the relevance of adequate nutrition in boosting immune function by supporting the innate and adaptive immunity systems of the body (18, 19). Nutrition supports innate immunity through the development and maintenance of physical barriers; production of antimicrobial proteins; growth, differentiation and chemotaxis of innate cells. It also helps in mediating the phagocytic and killing activities of neutrophils and macrophages as well as encourages the promotion of and recovery from inflammation (12, 19). On the other hand, nutrition supports the adaptive immune system by enhancing lymphocyte differentiation, proliferation, and homing as well as anti-viral cytokine production. It also supports antibody production; and the generation of memory cells which are essential antiviral defense mechanisms of the body (12, 19, 20). This is made possible by the presence of several vitamins, including vitamins A, B6, B12, C, D, E, and folate; and trace elements, including zinc, iron, selenium, magnesium, and copper; most of which can be gotten from adequate nutrition (12).

Apart from boosting the immune system for an effective immune response, some edible food substances such as *H. cordata* (21), green tea (22), *Ulva lactuca (U. lactuca)* (23), *Glycyrrhiza uralensis (G. uralensis)* (20) have been shown to possess *in vitro* virucidal and virus-inhibitory effects on human pathogenic viruses (24, 25). The observed antiviral effects of these food substances may be associated with their constituent phytochemicals, micronutrients and vitamins (14, 21, 26–29).

Presently, a number of synthetic antiviral medications are been used in the treatment of viral diseases for which a good number have shown promising results (27). Among these include lopinavir, arbidol, hydroxychloroquine and choloroquine phosphate, nucleoside analogs, neuraminidase inhibitors, and azithromycin (15, 30). However, there are still certain viral strains against which effective antiviral vaccines/medication have not yet being produced; especially viral strains belonging to the coronavirus family of viruses (27, 30). In addition to this, limited antiviral medications exist for combating certain respiratory viral infections including those caused by the human RSV (6). Incidences of drug resistant strains of different respiratory viruses makes it necessary to continually search for new therapeutics to combat respiratory viral infections (31, 32). There is thus a need to delve into research that would identify safer and more effective antiviral agents from natural products in order to augment the existing antiviral medications.

The prevalence and severity of different diseases is known to vary across different geographical regions and human populations (33, 34). Such difference has been attributed to factors including climatic, socioeconomic, political, and environmental factors (33, 34). Recent evidences are suggesting that nutrition plays important roles in the transmission and severity of viral infections (35, 36). We were interested in determining whether plant-derived food substances or their products would alter the course of respiratory virus's infection as well as understand possible mechanism involved in such effect. We reasoned that the presence of some vitamins and phytochemicals in foods may both boost the body's immunity to respiratory virus infection as well as suppress the proliferations of respiratory viruses. This systematic review is therefore an attempt to identify plant-derived edible food substances or their constituents, demonstrated to be active against viruses that infect human respiratory tracts as well as report potential mechanisms involved in such antiviral effects. To our knowledge, there is no prior systematic review on the antiviral efficacy of edible plant substances on respiratory viruses. Availability of this information will bring to light previously untapped antiviral benefits of plant-derived food substances thereby facilitating the development of additional layer of defense against respiratory viral infections.

## Method

**Figure 1:**
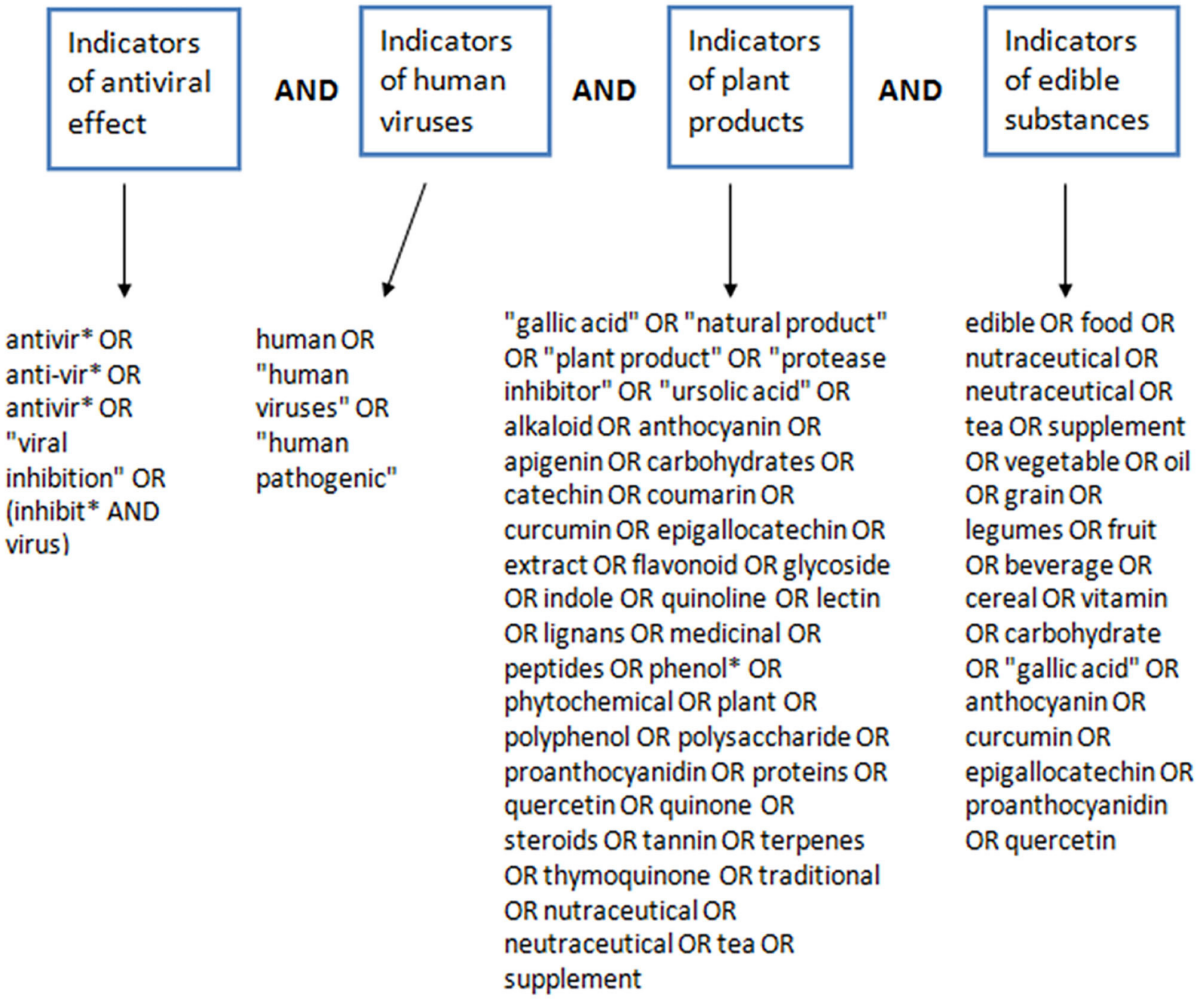
Construction of the search strategy for retrieval of literature on PDFGS with antiviral potentials.

This systematic review was aligned with the Preferred Reporting Items for Systematic Reviews and Meta-Analyses (PRISMA) guidelines (37). PUBMED and SCOPUS databases were searched systematically between April and May 2020, for articles that investigated the antiviral effects of natural products against human pathogenic viruses. Literature search was conducted independently by two of the authors (FU, BE-E) across the two databases. The database search covered all available publications from inception to the date of search. The search strategy used in the retrieval of literature for this review included different search terms that denote antiviral properties, human pathogenic viruses, natural products and edible substances. Search terms used to designate antiviral properties included “viral inhibition,” “inhibit virus,” “antiviral,” “antivirus.” Search terms used to designate human pathogenic viruses included “human virus,” “virus,” “human pathogenic.” Search terms used to designate edible, plant-based or natural products included “anthocyanin,” “apigenin,” “beverage,” “carbohydrate,” “catechin,” “cereal,” “coumarin,” “curcumin,” “edible,” “epigallocatechin,” “extract,” “flavonoid,” “food,” “fruit,” “gallic acid,” “glycoside,” “grain,” “indole,” “lectin,” “legumes,” “lignans,” “medicinal plant,” “medicinal,” “natural product,” “neutraceutical,” “nutraceutical,” “oil,” “peptides,” “phenol^*^,” “phytochemical,” “plant extract,” “plant product,” “plant,” “polyphenol,” “polysaccharide,” “proanthocyanidin,” “protease inhibitor,” “proteins,” “quercetin,” “quinoline,” “quinone,” “steroids,” “supplement,” “tannin,” “tea,” “terpenes,” “thymoquinone,” “traditional,” “ursolic acid,” “vegetable,” “vitamin,” “alkaloid.” These search terms were combined together in a search engine dependent boolean format to give the complete search strategy used for retrieving the literature used in this review. The construction of the search strategy (Figure 1) and the detailed strategy used to search PUBMED and SCOPUS is detailed below.

## Scopus

((TITLE-ABS-KEY (antivir^*^ OR anti-vir^*^ OR antivir^*^ OR “viral inhibition” OR (inhibit^*^ AND virus)) AND TITLE-ABS (human OR “human viruses” OR “human pathogenic”))) AND (TITLE-ABS (“gallic acid” OR “natural product” OR “plant product” OR “protease inhibitor” OR “ursolic acid” OR alkaloid OR anthocyanin OR apigenin OR carbohydrates OR catechin OR coumarin OR curcumin OR epigallocatechin OR extract OR flavonoid OR glycoside OR indole OR quinoline OR lectin OR lignans OR medicinal OR peptides OR phenol^*^ OR phytochemical OR plant OR polyphenol OR polysaccharide OR proanthocyanidin OR proteins OR quercetin OR quinone OR steroids OR tannin OR terpenes OR thymoquinone OR traditional OR nutraceutical OR neutraceutical OR tea OR supplement)) AND TITLE-ABS (edible OR food OR nutraceutical OR neutraceutical OR tea OR supplement OR vegetable OR grain OR legumes OR fruit OR beverage OR cereal OR vitamin OR carbohydrate OR gallic OR anthocyanin OR curcumin OR epigallocatechin OR proanthocyanidin OR quercetin OR dietary OR spice OR additives).

## Pubmed

((((antivir^*^[ti] OR anti-vir^*^[ti] OR antivir^*^[ti] OR “viral inhibition”[ti] OR (inhibit^*^[ti] AND virus[ti]))) AND (human OR “human viruses” OR “human pathogenic”)) AND (“gallic acid” OR “natural product” OR “plant product” OR “protease inhibitor” OR “ursolic acid” OR alkaloid OR anthocyanin OR apigenin OR carbohydrates OR catechin OR coumarin OR curcumin OR epigallocatechin OR extract OR flavonoid OR glycoside OR indole OR quinoline OR lectin OR lignans OR medicinal OR peptides OR phenol^*^ OR phytochemical OR plant OR polyphenol OR polysaccharide OR proanthocyanidin OR proteins OR quercetin OR quinone OR steroids OR tannin OR terpenes OR thymoquinone OR traditional OR nutraceutical OR neutraceutical OR tea OR supplement)) AND (edible OR food OR nutraceutical OR neutraceutical OR tea OR supplement OR vegetable OR oil OR grain OR legumes OR fruit OR beverage OR cereal OR vitamin OR carbohydrate OR “gallic acid” OR anthocyanin OR curcumin OR epigallocatechin OR proanthocyanidin OR quercetin).

### Eligibility Criteria

Studies included in this review were those that either reported on viruses that infects human or on surrogate of human viruses adapted for different experimental models. The human viruses considered were those that belong to any of the classes of viruses known to infect the human respiratory tract including influenza viruses, RSV, parainfluenza viruses (PIVs), metapneumovirus, coronavirus, respiratory AdV, RV, bocavirus, enterovirus, and parechovirus (2, 6, 7). Only studies done on animals, humans or cell lines, those that utilized constituents of edible foods as an antiviral intervention agent and those that reported some forms of outcome following treatment of virus-infected models with the intervention agents were considered for inclusion. Extracts obtained from non-edible plant parts were excluded. Studies that did not report a comparative untreated group were excluded from the review. There was no restriction on the year of publication or location of study. Only original research articles published in English language whose full texts are accessible online were included.

### Selection of Relevant Article

The titles and abstract of all the articles retrieved from each of the databases were screened against the aforementioned criteria. The selected articles were pooled together and duplicate entries were removed manually. The full texts of these selected articles were subsequently retrieved and subjected to full text screening for eligibility. Eligible articles were screened independently by two authors (FU, BE-E). The list of selected eligible articles, obtained by the two authors, were compared, and collated. In cases of differing opinion on the eligibility of any particular article, the article in question was discussed amongst the authors until a consensus was reached. Where the edibility status of a particular intervention agent is in doubt, additional literature search was conducted to establish whether such agent was edible or whether they are commonly found in edible foods.

### Assessment of the Quality of Selected Studies

The quality of the *in vitro* studies used for this review was assessed using an adapted version of the CONSORT statement similar to what was reported by Seyedpour et al. (38). Each of the included study was assessed whether it reported each of the 21 items of the study assessment checklist. They are assigned either a “yes” or “no” value for each items of the checklist depending on the authors' judgement. Assessment was done on the inclusion of relevant background information, statement of study's objectives as well availability of detailed information on the participants (particularly, the cell models and viruses). Availability of details on the virus strains, culture conditions, viability of the model organism (in the absence of viral infection) as well as evidence of successful viral infection following exposure of the virus to the model cells were also assessed. Availability of adequate information on the intervention agent (in this case, PDFSG) including its concentration and cytotoxicity were assessed. Reportage of methods for all intended outcomes, statement of sample sizes or numbers of repetition of experimental procedures, description of statistics used and appropriateness of the experimental methods/procedures to detect viral inhibitory effect were assessed for each included study. Each study was also assessed for the reportage of sufficient result for all outcomes investigated, demonstration of dose-graded effect, reportage of study limitation, and appropriate interpretation of observed result. Possible presence of sampling and detection bias was assessed by checking if the study reported randomization and blinding in their experimental procedures. Reportage of information on funding and potential conflict of interest were also assessed.

The quality of the included animal studies was assessed using the Systematic Review Center for Laboratory Animal Experimentation (SYRCLE) risk of bias tool (39). Each of the study was screened against a 9 item checklist and was assigned either a “high,” “low,” or “uncertain” risk depending on the outcome of the assessment by the authors.

### Data Extraction

Each of the eligible articles was read independently by two of the authors (FU, BE-E). The authors extracted a previously agreed set of relevant information from each of the articles under review and completed a predesigned form. The extracted information included the intervention antiviral agent, sources of the agent, test virus, experimental model, assay methodology for antiviral effects, antiviral concentrations, cytotoxic concentrations, antiviral mechanism (where available), author's information. The extracted information was reviewed by all the authors and was used to complete the summary table.

## Result and Discussion

**Figure 2:**
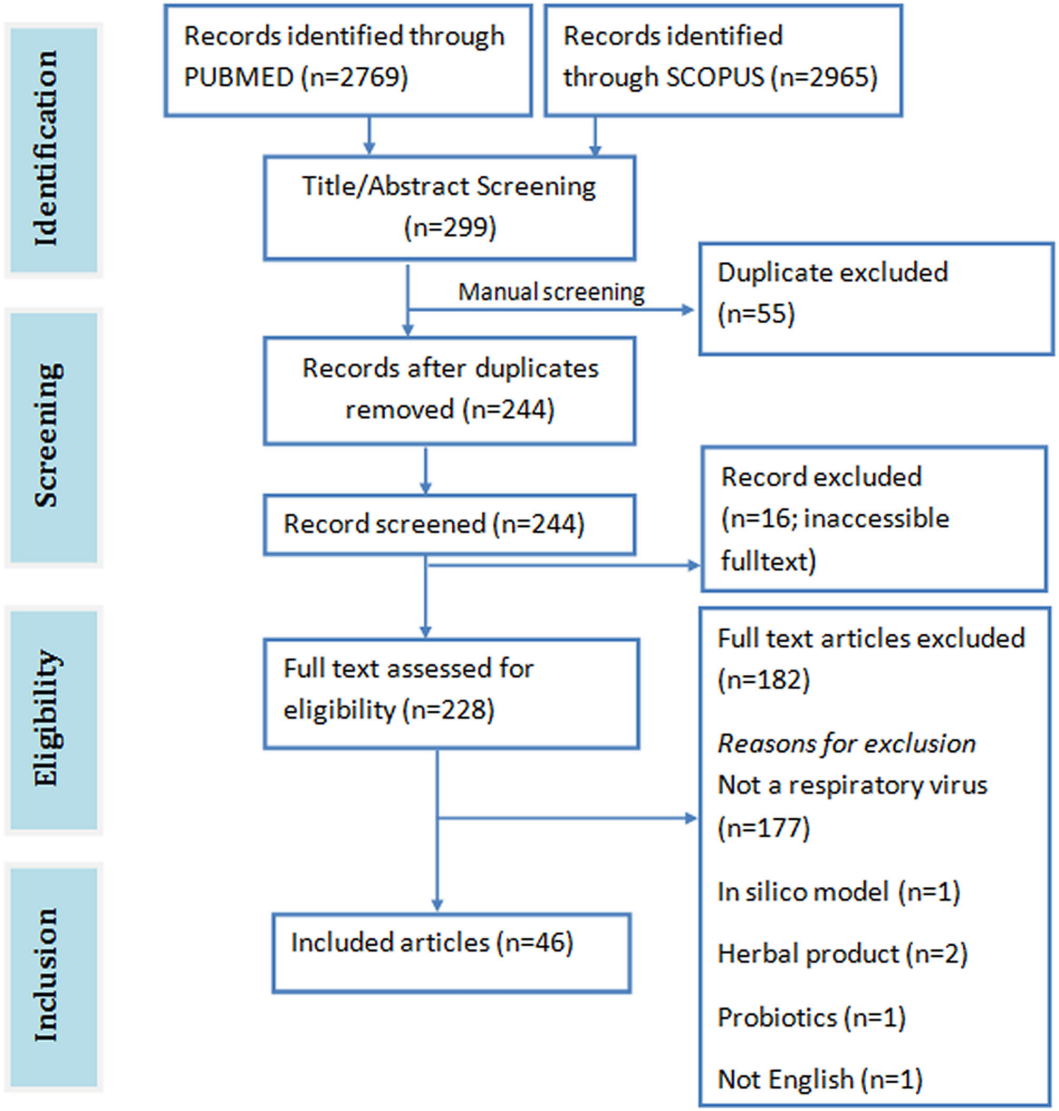
PRISMA flowchart of included studies.

A total of 5,734 papers were retrieved from both SCOPUS (2965) and PUBMED (2769) databases. Abstract and title screening yielded 299 publications (PUBMED: 193, SCOPUS: 106) with 244 unique articles after removing duplicates. Of these articles, only 228 were downloaded. The full text of the remaining 16 articles could not be accessed online. Forty (40) eligible articles were identified after screening the full text of the downloaded articles against the eligibility criteria (Figure 2). Forty-one (41) of the included studies were on cell lines, 11 were on mice, and 1 was on chicken egg.

### Outcome of Study Quality Evaluation

**Figure 3:**
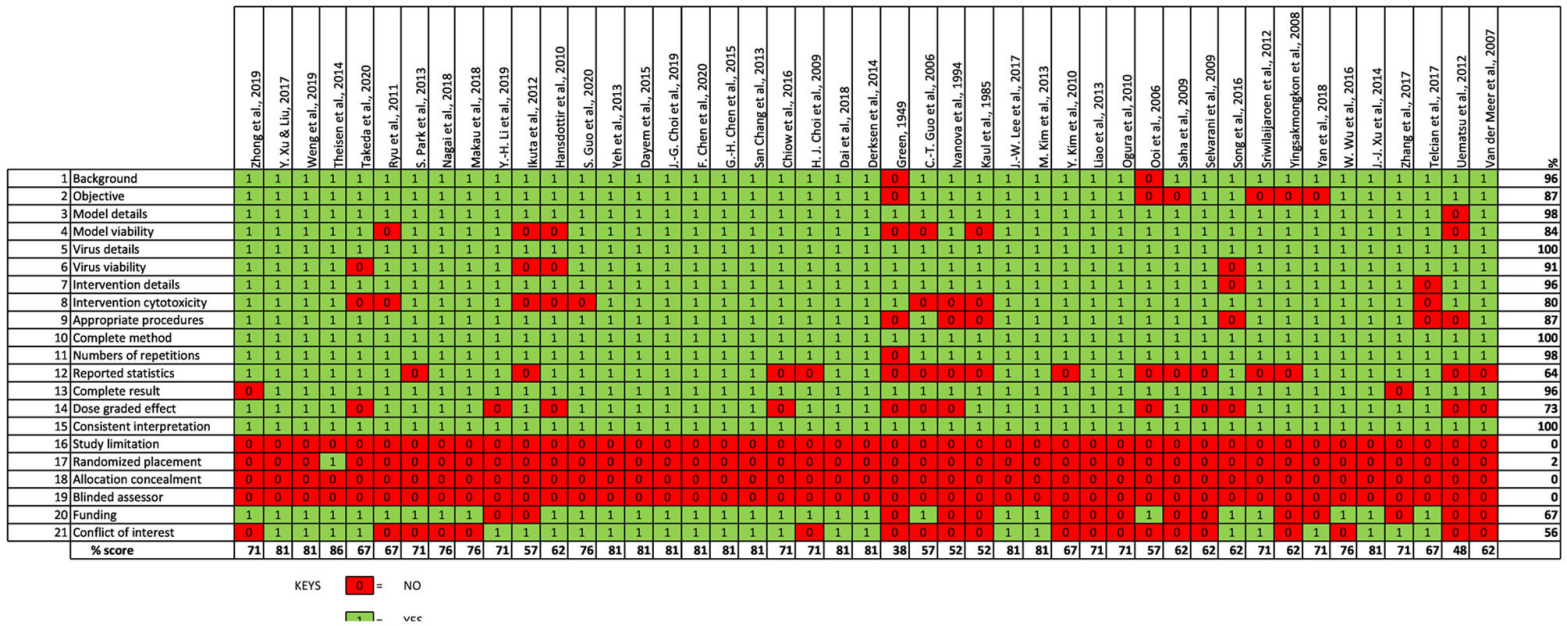
Quality assessment of included *in vitro* studies.

The findings from our evaluation of the quality of the included *in vitro* studies is shown in Figure 3. Most of the included studies provided sufficient information on study's background and objectives. Almost all the included studies provided adequate description of the virus strain, test cell line model and intervention agent (that is, PDFGS). However, only 84% of the included studies demonstrated the viability of the virus in the experimental models, 80% of the studies gave information on the cytotoxicity of the intervention agent to the un-infected cell model. Majority (87%) of the included studies used appropriate procedure to show the viral inhibition, provided a complete description of the methods used to estimate all intended outcome (100%), reported the numbers of repetition carried out for each effects estimate (98%), as well as reported outcome for all the investigated parameters (96%). Only about half of the studies presented statistical description in the methodology (64%). Dose-graded effect was reported in 73% of the included studies. All the included studies gave an interpretation consistent with their results.

None of the studies reported a study limitation. Just one of the included studies reported a form of randomization in the experimental procedure. None of the included study reported blinding or concealing the allocation from the investigator. Information on study funding and potential conflict of interest were reported by 67 and 56% of the included studies, respectively. Overall, the included studies was scored an average of 70% from a possible 100%.

**Figure 4:**
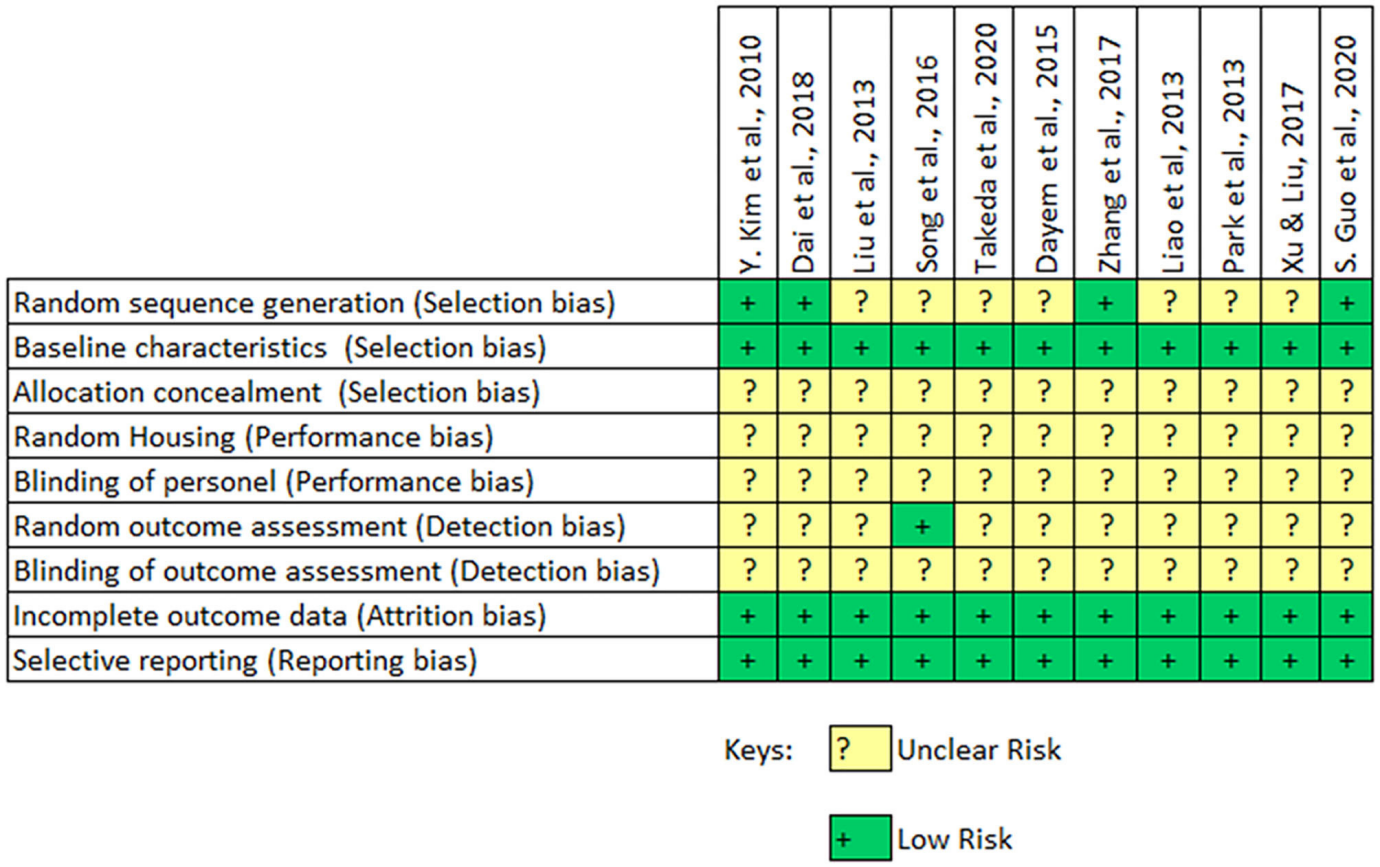
SYRCLE risk of bias assessment of included animal studies.

The findings following the assessment of the study quality of the included *in vivo* studies is shown in Figure 4. The findings indicate that the included studies had an unclear and potentially significant risk of selection bias. Only 4 out of the 11 studies carried out a randomization of their experimental setup. All the included studies provide sufficient baseline information on the experimental animal and experimental conditions. But none of the studies reported a concealment of allocations from the investigator. The included animal studies had an unclear risk of performance bias since none of them reported a random housing of the animals or the blinding of the caregiver. An unclear risk of detection bias was also observed in the included animal studies. However, the included studies had a low risk of attrition or reporting bias. The overall assessment indicates that the included studies had a potentially high risk of selection, performance and detection bias.

#### PDFGS Were Effective Against Different Strains of Respiratory Viruses

Some strains of human pathogenic influenza A virus (IAV), influenza B virus (IBV), RSV, human parainfluenza virus (hPIV), human coronaviruses, RVs and AdV are susceptible to different PDFGS (Table 1). Majority of the included studies investigated the antiviral effect of PDFGS on different subtypes of IAV, including H1N1 strains such as influenza A/Puerto Rico/8/34 (67, 70), influenza A/Jiangsu/1/2009 (71), influenza A/Fort Monmouth/1/1947 (68), and Amantadine resistant influenza A/WSN/33/S31N (60); H3N2 strains such as influenza A/Memphis/1/71, influenza A/Aichi/2/68 (67), and influenza A/JiangXi/312/2006 (68); H2N2 subtypes (58); H9N2 subtypes (10, 40) and H5N1 subtypes (10). Strains of IBV such as B/Nagasaki/1/87, B/Shanghai/261/2002 (16), and influenza B/human/Hubei/1/2007 (60) were also susceptible to PDFGS. Other susceptible respiratory viruses reported in this review included human coronavirus NL63 (HCoV-NL63) (44), RSV (A2, Long and B strains) (20, 59, 69), human parainfluenza virus type 2 (hPIV-2 Toshiba strain) (73), human parainfluenza virus type 3 (hPIV-3) (58), mouse hepatitis virus (21), rhinovirus 1B (RV1B) (72), AdV (type 5 prototype strain) (48).

**Table 1:** Summary table of included *in vitro* studies.

**Antiviral PDFGS**	**Sources**	**Susceptible Virus/host organism**	**Antiviral effect**	**Effective antiviral concentration, cytotoxicity, and selectivity index**	**Antiviral targets**	**References**
DMO-CAP	*Artemisia rupestris*	IAV (H1N1, H3N2)/MDCK and RAW 264.7 cells	CPE reduction (IC50)	31.78–42.91 μM (CC50 = 223 μM) SI = 7.02, 5.20	Viral replication, Viral-induced cytotoxicity, Host defense	(42)
Curcumin		IAV (H1N1)/human macrophage	Significant reduction in different cytokines	80 μM, no effect on macrophage viability.	Viral-induced cytotoxicity	(43)
Gallic, Chlorogenic, Caffeic acid		HCoV-NL63/LLC-MK2	Plague reduction (IC50)	Gallic: 71.48μM (CC50 > 500 μM) Chlorogenic: 43.45 μM (CC50 > 500 μM) Caffeic: 3.54 μM (CC50 > 500 μM)	Viral-host interaction	(44)
*Hamamelis virginiana* tannins extracts, Polyphenols (Gallic acid, Pentagalloylglucose, tannic acid, EGCG)	*Hamamelis virginiana*	IAV (H1N1)/A549	GFP reduction (IC50)	Extract: 1.1–36.2 μg/ml (CC50 = 223–968 μg/ml) Polyphenols: 5.6–18.3 μg/ml (CC50 144–733 μg/ml) SI = 15.2–85	Virus-host interaction, Viral replication	(41)
*Hibiscus sabdariffa* extract	*Hibiscus sabdariffa*	IAV (H1N1)/MDCK	–	–	Virus-host interaction	(45)
Phlorotannins	Brown Alga Ecklonia	IAV (H1N1, H3N2, H9N2)/MDCK cells	Neuraminidases inhibition (IC50)	4.5–41 μM	Viral replication	(40)
*A. melanocarpa* extract, Ellagic acid, myricetin	Black chokeberry	IAV (pH1N1, H1N1, H3N2), IBV/MDCK	Plague reduction (60%)	Plague reduction: 0.625 mg/ml	Viral replication	(46)
Adlay tea	Adlay seeds, barley seeds, soybeans and cassia seeds	IAV (H1N1, H3N2) IBV/MDCK	Plaque reduction (IC50)	2.11–5.13 mg/ml (CC50 > 40 mg/ml) SI > 8, SI > 19	Virus-host interaction	(16)
Peanut skin extract, Resveratrol	*Arachis hypogaea L*	IAV (H1N1, H3N2), IBV/MDCK	Plaque reduction (IC50)	Peanut skin: 1.3–3.2 μg/ml (CC50 = 5.4–9.1 μg/ml) SI = 3.6–7.4 Resveratrol: 5 μg/ml (CC50 > 35 μg/ml)	Early stage	(47)
*Portulaca oleracea* L. Extract	*Portulaca oleracea*	IAV (H1N1, H3N2)/MDCK	Plaque reduction (EC50)	112–220 μg/ml (CC50 = 8,067 μg/ml) SI = 36–71	Virus-host interaction, Viral replication, Early stage	(25)
Blackcurrant extract	Blackcurrant	RSV, IAV, AdV, and IBV/HEp-2, MDCK	Plaque reduction (IC50)	0.13–2.54% of blackcurrant extract	Virus-host interaction	(48)
Vit D		RSV A2/HTBE cells	–	–	Virus-induced cytotoxicity	(49)
Iridoid glycosides	*Fructus Gardeniae*	IAV (H1N1)/MDCK	Replication reduction (58%)	320 μg/ml	Viral replication, Virus- induced cytotoxicty	(50)
Glycyrrhizin, 18β-GA	*G. uralensis* (18β-GA active component-a gut metabolic product of Glycyrrhizin)	RSV (Long strain)/HEp-2, A549 cells	Plaque reduction (IC50)	4.3–4.5 μg/ml CC50 (71.5–76.3 μg/ml) SI = 15.9–26.8	Early stage, Virus -host interaction, Host defense	(20)
Isorhamnetin, Quercetin, Kaempferol, Diosmetin, Eriodictyol		IAV/MDCK	CPE reduction (EC50)	23–115 μM (CC50 > 245 μM) SI = 2–12	Virus-induced cytotoxicity, Viral replication, Virus-host interaction, early stage, post-infection	(51)
*G. thunbergii* extract, Geraniin (an ellagitannin)	*G. thunbergii*	IAV (H3N2, H2N1), IBV/MDCK	Neuraminidase Inhibition (IC50)	10.9–135 μg/ml (100% cell viability at 400 μg/ml)	Viral protein synthesis, Viral replication, post-infection	(52)
Isocorilagin (an ellagitannin)	*Canarium album*	IAV (H3N2, H2N1), IBV/MDCK	Plaque reduction (IC50)	4.64–23.7 μM (CC50 = 263.3 μM) SI = 11.1–56.75	Viral replication, Viral protein synthesis, post-infection	(53)
Ellagic acid	Strictinin, Pu'er tea	IAV (H1N1)/MDCK	Plaque reduction (50%)	Ellagic acid: 6 μM	Viral replication	(54)
*Z. officinale* extract	*Z. officinale*	RSV (long strain)/HEp-2 and A549	Plaque reduction (IC50)	73.3–144.9 μg/ml (CC50 = 1893.8 μg/ml) SI = 13.15	Virus-host interaction, Virus-induced cytotoxicity, Viral replication	(19)
Quercetin, *H. cordata* extract	*H. cordata*	MHV (Surrogate for coronavirus)/CCL9.1 cells	Plaque reduction (IC50)	Quercetin: 125 μg/ml (CC50 = 116 μg/ml) SI = 0.93 *Houttuynia*: 0.98 μg/ml (CC50 > 3.91 μg/ml) SI > 4	-	(21)
Quercetin 3-rhamnoside	*H. cordata*	IAV/MDCK	CPE reduction (65%)	10 μg/ml (95% viable at 100 μg/ml)	Viral replication	(28)
Curcumin	Curcuma longa L	IAV (H1N1, H9N2,H5N1,H3N2)/MDCK	Plaque reduction (EC50)	21.36 μg/ml (CC50 = 140.67 μg/ml) SI = 12.88	Viral replication, Viral protein synthesis, Virus-host interaction, Virus-induced cytotoxicity	(10)
*R. acetosa* Extract, EGCG, Procyanidin B2 digallate	*R. acetosa*	IAV (H1N1)/MDCK	Rumex Extract: Plaque reduction (68%) EGCG: Plaque reduction (80%) Procyanidin: Plaque reduction (80%)	Rumex Extract: 0.1 μg/ml (CC50 = 80 μg/ml) SI = 32, 36 EGCG: 2 μM (CC50 > 200 μM) Procyanidin: 0.2 μM (CC50 = 191 μM)	Viral-host interaction	(55)
Black tea	Black Tea	IAV/Chicken egg	Heamagglutination inhibition (IC50)	0.41–2 mg/egg		(56)
Edible bird's nest (EBN) extract	Edible bird's nest	IAV/(H1N1, H3N2)/MDCK	CPE reduction (IC50)	36–464 μg/ml	Virus-host interaction	(57)
Polysaccharide extract	Marine Algae (*Ulva lactuca*)	IAV/(H1N1, H2N2,H3N2)/MDCK	Heamagglutination inhibition (MIC50)	0.12–0.5 μg/ml SI = 8, 16	Virus-host interaction	(23)
Hesperetin, Quercetin		hPIV-3, RSV (long)/HEp-2	Plague reduction (76–97%)	200 μM (No cytotoxicity at 200 μM)	Viral replication, Viral-host interaction	(58)
Grape seed proanthocyanidin (GSP)	Grape Seed	RSV A2/A549	–	(No cytotoxicity at 5–10 μg/ml)	Viral replication, Viral protein synthesis, Virus-induced cytotoxicity	(59)
EGCG	Green tea	IAV (H1N1, H3N2), IBV/MDCK	CPE reduction (EC50)	5.7–17.3 μM (CC50 > 60, 195 μM) SI = 19.6	Viral replication, Viral-host interaction,	(22)
Isoquercetin, Quercetin, Fisetin, resveratrol, EGCG		IAV (H1N1, H3N2), IBV/MDCK	CPE reduction (EC50)	1.2–48 μM (TD50 45–200 μM) SI = 1.5–38	Viral replication, Viral protein synthesis, Virus-induced cytotoxicity	(29)
Germacrone	*Rhizoma Curcuma*	IAV (H1N1, H3N2), IBV/MDCK, A549	MDCK: CPE reduction (EC50) A549: CPE reduction (EC50)	MDCK: 3.82–7.12 μM (CC50 > 250 μM) SI > 41 A549: 3.82–7.12 μM (CC50 > 250 μM) SI > 93.9	Viral replication, Viral protein synthesis, Virus-host interaction	(60)
Sulphated polysaccharide extract	Edible blue-green algae (*Aphanothece sacrum*)	IAV(H1N1)/MDCK	Plague reduction (IC50)	1.2 μg/ml (CC50 = 7,100 μg/ml) SI = 6,200	Virus-host interaction	(61)
3,4-dicaffeoylquinic acid and 3,5-dicaffeoylquinic acid	*Youngia japonica*	RSV (Long strain)/Human epithelial type 2 (HEp-2) cells	CPE reduction (IC50)	0.5 μg/ml (No cytotoxicity up to 100 μg/ml)	Viral replication	(62)
Glycosyl Hesperidin		IAV (H1N1, H3N2)/MDCK	Viral titer reduction (32–60%)	12 mM (No cytotoxicity from 0 to 25 mM)	Viral replication	(63)
Extracts from *A. digitata* fruit, seed leaves	*A. digitata* (Baobab)	RSV/H-1 cells IAV (H3N2)/MDCK	CPE reduction (MIC50)	RSV: 16.2 μg/ml IAV: 0.12 μg/ml (cytotoxic at > 130 μg/ml)	Host defense	(64)
Acteoside (phenylpropanoid glycoside)	Kuding Tea	IAV (H1N1)/Primary lymphocytes	–	no cytotoxicity between 1.25 and 160 μM	Host defense	(65)
Green tea, Guava tea	Green tea leaves, Guava leaves	IAV (H1N1)/MDCK	Neuraminidase Inhibition (IC50)	Green tea: 0.25–1.44% Guava tea: 0.002–0.58% no cytotoxicity at 25%	Viral replication, Virus-host interaction	(66)
Lectin-like compounds	Japanese plum fruit	IAV (H3N2)/MDCK	Plague reduction (IC50)	0.53–6.35 μg/ml (1,000 μg/ml no cytotoxicity)	Viral-host interaction	(67)
Cirsimaritin	*Artemisia scoparia*	IAV (H3N2, H1N1)/MDCK	CPE reduction (IC50)	5.8–11 μg/ml (TC50 = 153 μg/ml) SI = 13.8–26.4	Viral replication, Viral protein synthesis, Virus-induced cytotoxicity	(68)
Tangeretin, polymethoxylated flavones extract	Guangcheni (*Citrus reticulata*)	RSV (A2, B)/HEp-2 cells	Tangeretin: Plague reduction (IC50) Extract: CPE reduction (IC50)	Tangeretin: 5.4–7 μM (CC50 = 375–413 uM) Extract: 6.7–15 μM (CC50 = 252–417 μM) SI = 16.4–62.3	Viral replication	(69)
Quercetin		IAV (H3N2, H1N1)/MDCK	CPE reduction (IC50)	2.74–7.76 μg/ml (No cytotoxicty at 250 μg/ml)	Viral replication, Viral protein synthesis, Virus-induced cytotoxicity	(70)
Geniposide	*Gardenia jasminoides* fruit	IAV (H1N1)/MDCK	MDCK: CPE reduction (IC50)	MDCK: 87.68 μmol/L (95% viable at 1,040 μmol/L)	Viral replication	(71)
Vit D, Cathelicidin		RV1B and RSV A2/HeLa Cells and BEAS-2B	Calcitriol: Viral release reduction (60%) Cathelicidin: Viral release reduction (80%)	RV1B: Calcitriol: 1,000 nmol RV1B:Cathelicidin: 100 μg/ml	Viral replication, Virus-induced cytotoxicity, Host defense	(72)
Lectins including concanavalin A, *Lens culinaris* agglutinin and peanut agglutinin		hPIV-2/LLCMK2 cells	–	–	Viral replication, Viral protein synthesis, Viral-host interaction	(73)
Urtica dioica agglutinin	Urtica dioica	MHV(Surrogate for coronavirus)/Mouse LR7 cells	Viral infection reduction (EC50)	0.53 μM (CC50 = 9.9 μM) SI = 18.68	Early stage	(74)

*18β-GA, 18β-glycyrrhetinic acid; A. digitata, Adansonia digitata; A. melanocarpa, Aronia melanocarpa; A549, human alveolar basal epithelial cells; CC50, 50% cytotoxic concentration; CPE, cytopathogenic effect; DMO-CAP, 6-demethoxy- 4′-O-methylcapillarisin; EC50, Concentration of an agent that gives half-maximal response; EGCG, Epigallocatechin gallate; G. thunbergii, Geranii thunbergii; G. uralensis, Glycyrrhiza uralensis; GFP, Green flourescent protein; H. cordata, Houttuynia cordata; HCoV-NL63, Human coronavirus NL63; HEp-2, Human epithelial type 2; hPIV-3, Human parainfluenza virus type 3; HTBE, Human tracheobronchial epithelial; IAV, influenza A virus; IBV, Influenza B virus; IC50, Half maximal inhibitory concentration; LLC-MK2, Rhesus Monkey Kidney Epithelial Cells; MDCK, Madin-Darby Canine Kidney; MHV, Murine hepatitis Virus; MIC50, Minimum Inhibitory Concentration required to inhibit the growth of 50% of organisms; R. acetosa, Rumex acetosa; RSV, Respiratory syncytial virus; TD50, Median toxic dose; Vit D, Vitamin D; Z. officinale, Zingiber officinale*.

#### Life Cycle of IAV

The evidence presented in this review indicates that PDFGS affected different stages in the lifecycle of respiratory viruses. A brief overview of the lifecycle of IAV and RSV is presented to serve as a background for subsequent discussion on the antiviral mechanism of these PDFGS, at each stages of respiratory virus's lifecycle. The choice of IAV and RSV is because majority of the evidence presented in this review are based on these two viruses.

**Figure 5:**
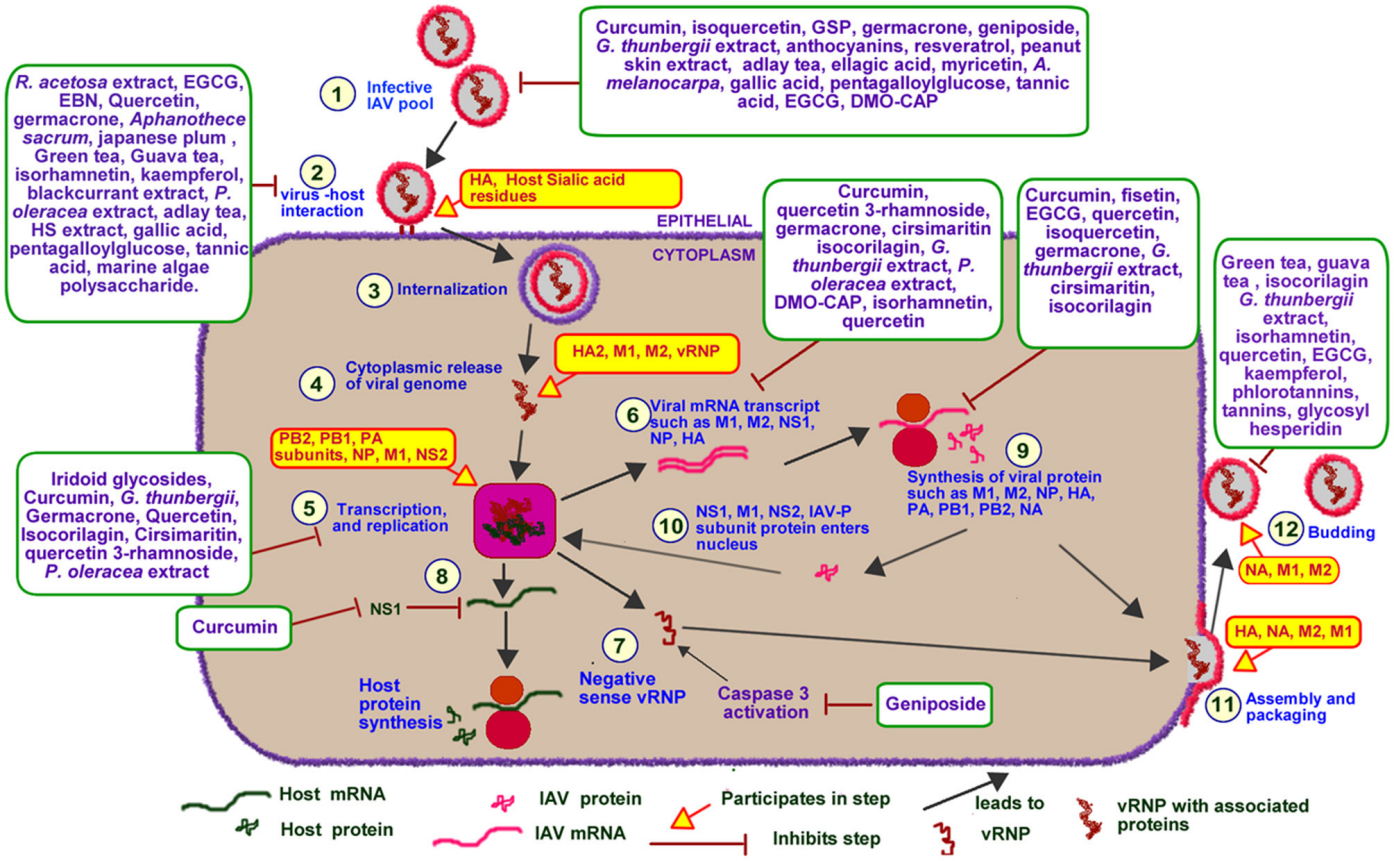
Effect of PDFGS on different stages of IAV lifecycle. Infective IAV enters cellular environment following exposure (1). It interacts with the sialic acid residues of host membrane glycoprotein using its HA protein (2). The virus is internalized into the cytoplasm in a clarithin-dependent and independent mechanism (3). Increased acidic ph of the endosome triggers the activation of M1, fusion of viral genome with the endosomal membrane and release of viral genome into the cytoplasm (4). The viral RNA enters the nucleus where it utilizes host enzymes and some viral proteins such as PA, PB1, PB2, and NP for replication and transcription of viral genome (5). This leads to the production of viral mRNA transcripts (6), viral full length negative sense viral RNA (7), and suppression of host mRNA and protein synthesis (8). The viral mRNA is translated with the host protein synthesis apparatus (9). Some of the synthesized viral proteins are exported to the nucleus to facilitate the transcription and replication of viral genome (10). The others are packaged alongside with viral RNA strands into infective IAV particles (11). The infective IAV buds off from the cell into extracellular spaces and perpetuate further viral infections (12). Different PDFGS as shown in the image, are able to reduce IAV cellular titer, inhibit IAV host interaction, IAV cytoplasmic release, IAV transcription and replication, IAV mRNA transcript and protein synthesis, IAV assembly, packaging, and budding. *A. melanocarpa, Aronia melanocarpa*; DMO-CAP, 6-demethoxy-4′-O-methylcapillarisin; EBN, edible bird nest; EGCG, Epigallocatechin gallate; *G. thunbergii, Geranii thunbergii*; GSP, Grape seed proanthocyanidin; HA, hemagglutinin; HA2, Fusion peptide; HS, *Hibiscus sabdariffa*; IAV-P, IAV polymerase; M1, Matrix protein 1; M2, matrix protein 2; NA, Neuraminidase; NP, nucleoprotein; NS1, Non-structural protein 1; NS2, Non-structural protein 2; *P. oleracea, Portulaca oleracea*; PA, Polymerase acidic protein; PB1, Polymerase basic protein 1; PB2, Polymerase basic protein 2; *R. acetosa, Rumex acetosa*; vRNP, Viral ribonucleoprotein.

An overview of IAV life cycle is illustrated in Figure 5. IAV infections are initiated following exposure to infectious IAV particles. Once an infective IAV particle gets into the respiratory tract environment, it utilizes its viral hemagglutinin (HA) protein to interact with sialic acid residues of membrane glycoproteins on host epithelial cells. Following successful host-virus interaction, IAV particles are taken up into the cell through endocytosis or macropinocytosis within an endosome (75). The low pH of the endosome induces a conformational change in IAV HA leading to the exposure of IAV fusion peptide (HA2) and subsequent fusion of the virus particle to the endosomal membrane (76, 77). The low pH also favors the activation of matrix protein 2 (M2) ion channel leading to the acidification of the viral core. This acidification leads to the dissociation of viral ribonucleoprotein (vRNP) from its associated matrix protein 1 (M1), as such, facilitating the cytoplasmic release and nuclear import of the vRNP (76, 77). IAV utilizes host nuclear import factors to facilitate the importation of vRNP into the nucleus (76).

IAV genome consists of an aggregate of eight individual, negative-sense vRNP complexes which encodes a total of 11 viral genes including neuraminidase (NA), M1, M2, HA, nucleoprotein (NP), non-structural protein 1 (NS1), non-structural protein 2 (NS2), polymerase acidic protein (PA), polymerase basic protein 1 (PB1), polymerase basic protein 2 (PB2), and polymerase basic protein 1–F2 (PB1-F2) (78). Each of the vRNP are wrapped around multiple copies of NP and are collectively held together by the heterotrimeric viral polymerase, consisting of the PB1, PB2, and PA (76, 78). The heterotrimeric viral RNA-dependent RNA polymerase catalyzes the replication of vRNP in a reaction that involves an initial synthesis of a complimentary RNA which serves as template for synthesis of the new viral ribonucleic acid (RNA) (76). IAV polymerases also produce messenger ribonucleic acid (mRNA) transcript from each of the vRNP. The presence of splice sites on some IAV mRNA transcripts triggers the host cell spliceosome to generate spliced transcripts such as those that encodes M2 and NS2 from IAV M and NS mRNA transcripts, respectively. The IAV mRNA transcripts are subsequently exported to the cytoplasm and are translated using both endoplasmic reticulum-associated and cytosolic host ribosomes (76). Some of the synthesized viral proteins such as NS1, NS2, and M1 are exported to the nucleus to facilitate the replication and transcription of viral genome (76). Other viral proteins such as NA, HA, and M2 are exported to the plasma membrane where they are used alongside with newly synthesized vRNPs to produce progeny IAV virions (76). The newly assembled IAV virons subsequently buds off from host cell in a process catalyzed by NA (76).

#### Life Cycle of RSV

**Figure 6:**
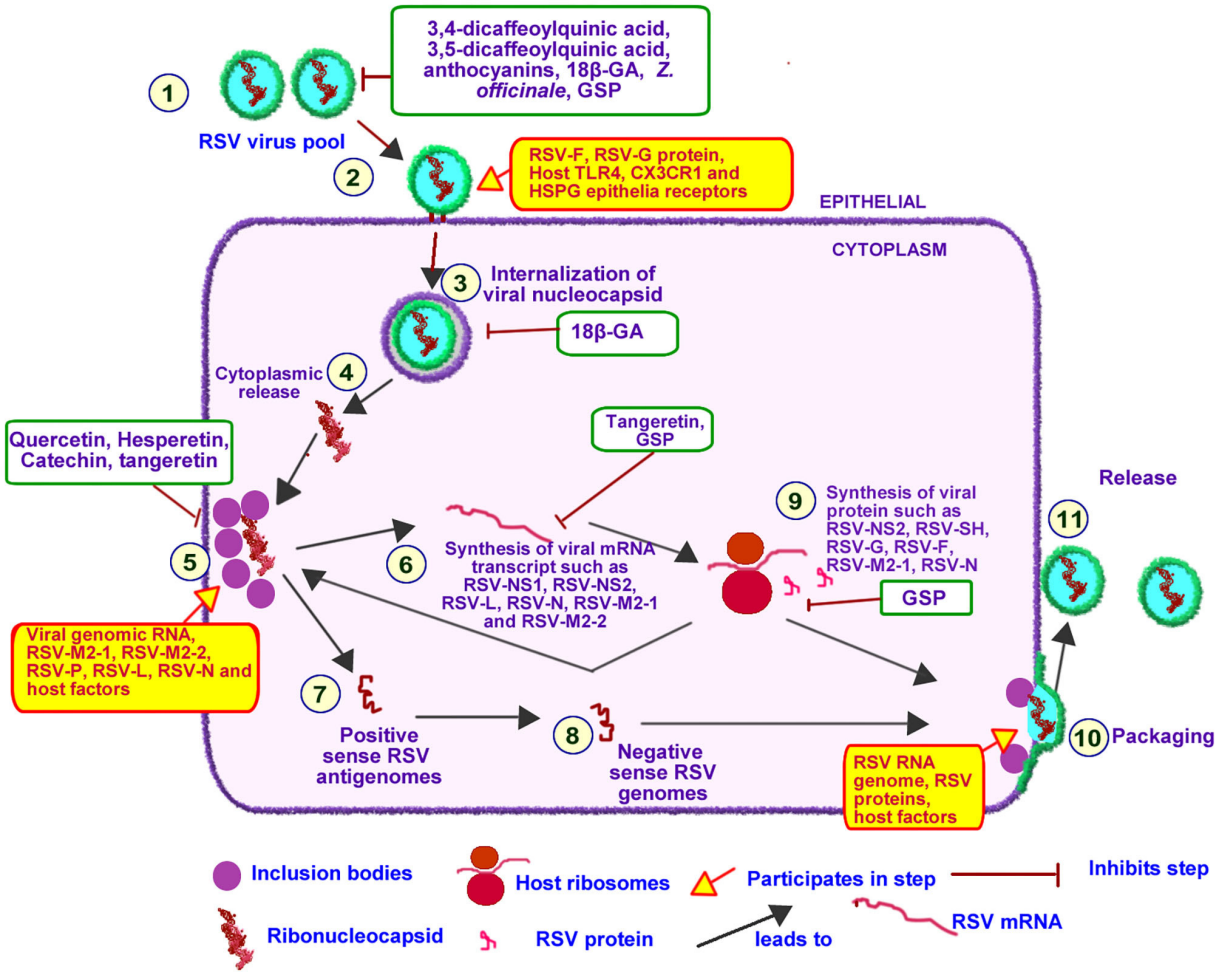
Effect of PDFGS on different stages of RSV lifecycle. Infective RSV gets into the respiratory tract following exposure to RSV viruses (1). RSV interacts with epithelial receptor proteins such as TLR4, CX3CR1 and HSPG using its RSV-F, RSV-G, and RSV-SH proteins (2). Intact RSV enters the host cytosol in an endosome (3). RSV nucleocapsid is released into the cytoplasm (5). Replication and transcription of RSV genome takes place at the inclusion bodies leading to the production of viral mRNA transcript (6) or full length positive sense antigenomes(7) following regulatory control by RSV M2-2. The positive sense antigenome is used as a template to generate the negative sense viral genome (8). The viral mRNA transcript is translated with the host mRNA translation apparatus (9). The negative sense viral RNA genome is packaged alongside with some viral proteins and other host factors required for RSV infectivity (10) this eventually buds off (11) into the pool of infective RSV. PDFGS reduced the cellular pool of infective RSV, suppressed RSV host receptor interaction as well as inhibited RSV internalization, replication, and transcriptional activities. PDFGS also lowered the levels of RSV mRNA transcripts as well as RSV protein. 18β-GA, 18β-glycyrrhetinic acid; CX3CR1, CX3C chemokine receptor 1; GSP, Grape seed proanthocyanidin; HSPG, Heparan sulfate proteoglycan; RSV, Respiratory syncytial virus; RSV-F, RSV fusion protein; RSV-G, Glycoprotein; RSV-L, RSV RNA-dependent RNA polymerase; RSV-M2-1, RSV matrix M2-1; RSV-M2-2, RSV matrix M2-2; RSV-N, RSV nucleoprotein; RSV-NS1, RSV non-structural protein 1; RSV-NS2, RSV non-structural protein 2; RSV-P, RSV phophoprotein; RSV-SH, RSV hydrophobic protein; TLR4, Toll-like receptor 4; *Z. officinale, Zingiber officinale*.

An overview of RSV life cycle is illustrated in Figure 6. RSV genome consist of a single stranded negative-sense RNA which codes for 11 viral proteins (79). RSV RNA-dependent RNA polymerase (RSV-L) encapsulates RSV RNA, RSV nucleoprotein (RSV-N) and RSV phophoprotein (RSV-P) to form the helical RSV ribonucleoprotein (RSV-RNP) complex (80). The transmembrane RSV glycoprotein (RSV-G), RSV fusion protein (RSV-F), and RSV hydrophobic protein (RSV-SH), localized on the surface coats of RSV, functions to attach of RSV to bronchial epithelial cell-resident receptor proteins such as, CX3C chemokine receptor 1, toll-like receptor 4, and heparan sulfate proteoglycan (80, 81).

Binding of RSV to host cell triggers the endocytosis and internalization of intact RSV into host cells (82). RSV-RNP subsequently associates with inclusion bodies which are localized near the cellular membranes and golgi apparatus (81, 82). The inclusion body-RSV-RNP complex forms active replication and transcription units of RSV since they contain viral proteins such as RSV-N, RSV matrix M2-1 (RSV-M2-1), RSV-L, RSV-P, and genomic RNA as well as other host factors (inherited from previously infected cells) required for the transcription and replication of RSV genome (81).

RSV-L can generate both RSV mRNA transcripts as well as synthesize full length positive sense antigenomes. The prevailing activity at any given time appears to be regulated by RSV matrix M2-2 (RSV-M2-2) protein which senses the pool of viral proteins (81). Accumulation of viral proteins favors the synthesis of full length positive sense RSV antigenome which forms a template for the synthesis of negative sense RSV genome (81). The generated RSV mRNA transcript are subsequently translated with host ribosome complex (81).

Some of the RSV proteins promote further proliferation and infectivity of RSV. For instance, RSV-N mediated inhibition of PKR leads to an unrestricted replication of RSV genome (81). RSV-N protein is known to interacts with MDA and RIG-1, within the inclusion bodies, hereby suppressing innate immune response to RSV (83). RSV non-structural protein 2 (RSV-NS2) have been reported to mediate the sloughing of infected and dead epithelial cells from the upper respiratory tract to the lower respiratory tract region hereby increasing RSV transmission to the lower respiratory tract (81).

#### PDFGS Suppressed IAV Host Entry

The data presented in this review showed that different PDFGS interfered with the different stages of IAV lifecycle (Figure 5). Several PDFGS interfered with IAV entry into host cells (**Table 4**). PDFGS such as edible bird nest (EBN) extracts, lectin-like compounds from Japanese plum, green tea, guava tea, isorhamnetin, tannins, HS, and polysaccharides from marine algae suppressed IAV-host cellular interactions through the inhibition of viral HA activities (41, 45, 51, 57, 66, 67). PDFGS such as *Portulaca oleracea (P. oleracea)* extracts, quercetin and EBN are able to prevent virus-host interaction by binding with strong affinity to the surface coats of IAV (25, 57, 70). Yingsakmongkon (Yingsakmongkon, 2008) reported that a lectin-like compound from Japanese plum which agglutinated pig erythrocytes, prevented virus-host interaction. By virtue of their structural characteristics, lectins can potentially interact with glycoprotein on the cell surface to inhibit viral entry (73). This possibility is supported by evidence from *in silico* studies which predicted a more stable interactions between *Galanthus nivalis* agglutinin-related lectins and sialic acid resides of host cells, than with IAV HA (84).

Some PDFGS like epigallocatechin gallate (EGCG) can damage viral surface coat, as such, preventing an efficient virus-host interactions (22). *In silico* analysis by Derksen et al. (55), predicted that procyanidin B2-di-gallate, procyanidin B2, EGCG, and epicatechin had good binding affinity to regions of IAV HA which is responsible for binding to host's sialic acids. The study predicted a better binding affinity for procyanidins that has increased levels of polymerization and galloylation. As such procyanidin B2-di-gallate was predicted to have a better binding affinity with IAV HA than procyanidin B2, EGCG and epicatechin (55). Other computational studies such as those by Ou et al. (85), predicted that curcumin can block the interactions between IAV HA and its cognate receptors by occupying regions on IAV HA responsible for its interaction with host sialic acid residues. Inhibition of viral entry will prevent further propagation of viruses, as such presents an attractive therapeutic target against viruses (70).

#### PDFGS Suppressed IAV Replication, Transcription, and Proliferation

The data presented in this review shows that certain PDFGS inhibited the proliferation of IAV by interfering with IAV transcription and replication (Table 2). PDFGS such as quercetin 3-rhamnoside, cirsimaritin, *P. oleracea*, and 6-demethoxy-4′-O-methylcapillarisin (DMO-CAP) were observed to suppress the generation of mRNA transcripts of IAV matrix protein (IAV-M) in IAV-infected cultured cells (25, 28, 42, 68). Quercetin, *Geranii thunbergii (G. thunbergii)* extract and isocorilagin were also shown to inhibit the expression of IAV HA mRNA transcripts in IAV infected MDCK cells (52, 53, 70). *G. thunbergii* and curcumin were also observed to suppress the levels of IAV NS1 mRNA transcript in *in-vitro* models (52, 60). Suppression of IAV polymerase activities were also reported by Guo et al. (50) following treatments of IAV exposed cells with iridoid glycosides. Suppression of IAV mRNA expression by these PDFGS is suggestive of an impaired IAV transcription and replication activities.

**Table 2:** Effect of PDFGS on viral replication.

**Agent**	Biomarker/effect^*^	**Susceptible virus**	**References**
Quercetin 3-rhamnoside	IAV-M↓	IAV	(28)
Curcumin	Viral replication ↓, Viral infectivity ↓, Lung viral titer ↓	IAV	(10)
Quercetin, Hesperetin, Catechin	Replication ↓, Infectivity ↓	hPIV-3, RSV	(58)
isoquercetin	Lung viral titer ↓	IAV, IBV	(29)
GSP	F mRNA ↓, RSV-N mRNA↓ Viral titer ↓	RSV	(59)
Germacrone	NP mRNA↓, Viral Titer ↓, NA activity ↓, lung Viral titer ↓	IAV, IBV	(60)
Curcumin	NS1 mRNA ↓	IAV	(86)
Green tea, Guava tea	NA activity ↓	IAV	(66)
Cirsimaritin	IAV-M protein mRNA ↓	IAV	(68)
Tangeretin	Viral mRNA (RSV-NS1, RSV-NS2, RSV-L, RSV-N, RSV-M2-1, and RSV-M2-2 forms) ↓ Viral replication (RSV-L) ↓	RSV	(69)
Concanavalin A, *lens culinaris* agglutinin and peanut agglutinin	NP mRNA ↓, tubules disruption (affecting virus release)↑, Viral release ↓	hPIV-2	(73)
Vit D, Cathelicidin	Viral RNA ↓, viral release ↓	RV1B	(72)
Geniposide	Viral lung titer ↓	IAV	(71)
Phenolic acid (Caffeic, Chlorogenic, Gallic)	Viral titer ↓	HCoV-NL63	(44)
Isocorilagin	NA activity ↓, HA mRNA ↓	IAV	(53)
Fresh *Z. officinale* extract	Viral titer ↓	RSV	(19)
Glycyrrhizin and 18β-GA	Viral titer ↓	RSV	(20)
Ellagic acid and gallic acid	Viral release ↓	IAV	(54)
*G. thunbergii* extract	Viral titer ↓,HA mRNA ↓, NS1 mRNA ↓, NA activity↓	IAV, IBV	(52)
Isorhamnetin	Lung virus titer ↓, viral mRNA ↓	IAV	(51)
Isorhamnetin, Quercetin, Kaempferol	NA activity↓	IAV	(51)
Anthocyanins	Viral titer ↓	RSV, IAV, IBV, ADV	(48)
*P. oleracea extract*	Viral titer ↓, M1 mRNA ↓	IAV	(25)
Peanut (*Arachis hypogaea L*.) skin extract, Resveratrol	Viral titer ↓	IAV,IBV	(47)
Adlay tea	Viral titer ↓, replication (time of addition assay) ↓	IAV, IBV	(16)
*A. melanocarpa* (black chokeberry) extract, ellagic acid, myricetin	Viral titer ↓, lung virus titer ↓	IAV, IBV	(46)
Phlorotannins	Viral titer ↓, NA activity ↓	IAV	(40)
Tannins (gallic acid, pentagalloylglucose, tannic acid, EGCG)	Viral titer ↓ NA activity ↓	IAV	(41)
DMO-CAP	Viral titer ↓, M2 mRNA ↓	IAV	(42)
Iridoid glycosides	IAV-P activity↓	IAV	(50)
EGCG	NA activity↓	IAV, IBV	(22)
Quercetin	HA mRNA ↓	IAV	(70)
Glycosyl Hesperidin	NA activity ↓	IAV	(63)
3,4-dicaffeoylquinic acid and 3,5-dicaffeoylquinic acid	Virus titer ↓	RSV	(62)

* *only effects that are significantly different from the control are shown; ↓Parameter is significantly lower than the untreated virally- infected model; ↑Parameter is significantly higher than the untreated virally- infected model*.

*A. melanocarpa, Aronia melanocarpa; DMO-CAP, 6-demethoxy-4′-O-methylcapillarisin; EGCG, Epigallocatechin gallate; F, Fusion protein; G. thunbergii, Geranii. thunbergii; GSP, Grape Seed Proanthocyanidin; HA, hemagglutinin; HCoV-NL63, Human coronavirus NL63; hPIV-2, Human parainfluenza virus type 2; hPIV-3, Human parainfluenza virus type 3; IAV, influenza A virus; IAV-M, IAV matrix protein; IAV-P, IAV polymerase; IBV, Influenza B virus; M1, matrix protein 1; NA, neuraminidase; NP, nucleoprotein; NS1, non-structural protein 1; P. oleracea, Portulaca oleracea; RSV, Respiratory syncytial virus; RSV-L, RSV RNA-dependent RNA polymerase; RSV-L, RSV RNA-dependent RNA polymerase; RSV-M2-1:RSV matrix M2-1; RSV-M2-2, RSV matrix M2-2; RSV-N, RSV nucleoprotein; RSV-NS1, RSV non-structural protein 1; RSV-NS2, RSV non-structural protein 2; RV1B, rhinovirus 1B; Vit D, Vitamin D; Z. officinale, Zingiber officinale*.

Evidence from this review further showed that PDFGS such as germacrone, green tea, guava tea, isocorilagin, *G. thunbergii*, isorhamnetin, quercetin, kaempferol, glycosyl hesperidin, EGCG, gallic acid, pentagalloylglucose, and tannic acid inhibited IAV NA activities in infected cell lines (41, 51–53, 60, 63, 66). Inhibition of viral NA activities could lead to, or suggest an impaired release of infective progeny IAV virions (87) as well as limits IAV's ability to infect new cells by interfering with the attachment of IAV to host cells (88, 89). Reduction in IAV NA activities following treatments with phlorotannins and tannins have been associated with concomitant reduction in IAV titer (40, 41).

Inhibition of IAV NA by flavonoids is further supported by evidences from molecular docking stimulations. Such computational studies predicted strong inhibitory binding affinity between different flavonoids and the active sites of IAV NA (90–93). For instance, computational studies by Kannan and Kolandaivel (2018) predicted a stronger interaction between IAV NA and cyanidin-3-sambubiocide, than between oseltamivir-and IAV NA. Theaflavin found in tea leaves, was also predicted to have high inhibitory interaction with amino acids of IAV NA (94). Molecular docking studies by Chen et al. (53), predicted the potentials of isocorilagin to bind to highly conserved regions of IAV NA as such making it potentially effective against multiple strains of IAV including those resistant to existing IAV NA inhibitors.

Inhibition of IAV NA is an important therapeutic target in management of IAV infection. Oseltamivir is a commonly prescribed antiviral agent whose mode of action is based on its ability to inhibit NA activities (28). Other PDFGS shown to reduce IAV titer in experimental models of IAV infection includes DMO-CAP, *Aronia melanocarpa* (*A. melanocarpa*) extracts, ellagic acids, myricetin, adlay tea, peanut skin extract, resveratrol, *P. oleracea*, anthocyanin, isorhamnetin, gallic acid, geniposide, isoquercetin, and curcumin (16, 25, 29, 42, 46–48, 51, 54, 71). Taken together, these pieces of evidence show that PDFGS are capable of suppressing IAV proliferations *in vitro*.

#### PDFGS Suppressed the Synthesis of IAV Proteins

Treatments of IAV infected cells with some PDFGS were observed to suppress the expression of different IAV protein (Table 3). The reduction in the levels of viral proteins following treatments of IAV infected cells with PDFGS suggests that these PDFGS either inhibited the synthesis of such proteins or inhibited IAV proliferation. This is evident from studies that associated the reduction in IAV protein synthesis with a reduced IAV titers, following treatments with germacrone, *G. thunbergii* extract, and isoquercetin (29, 52, 60). IAV-infected cells that were treated with PDFGS such as germacrone, quercetin, *G. thunbergii extract* and isocorilagin reduced the levels of IAV NP (29, 52). Viral NP functions in protecting viral genomes, facilitating viral entry, evasion of host immune responses and in the expression of viral genes (95). NP also appears to be an elongation factor for the activity of viral RNA polymerase which functions in the replication of the viral RNA genome (96). Suppression of viral NP by PDFGS would therefore impair several viral functions including viral replication, hereby leading to reduction in viral load. Treatments of IAV infected cells with EGCG, quercetin, isoquercetin and *G. thunbergii* extracts suppressed the levels of HA protein (29, 52). Reduction in HA protein levels would impair the assembly and budding processes of progeny IAV virions (76). Reductions in HA is also potentially capable of impairing the interactions between IAV and host glycoprotein receptors (76).

**Table 3:** Effect of PDFGS on viral protein synthesis.

**Agent**	Biomarker/Effect^*^	**Viruses**	**References**
Curcumin	M2↓	IAV	(10)
Fisetin	M1↓	IAV	(29)
EGCG, Quercetin, Isoquercetin	M1↓, HA↓	IAV	(29)
GSP	F protein ↓	RSV	(59)
Germacrone	NP ↓	IAV, IBV	(60)
Concanavalin A, lens culinaris agglutinin and peanut agglutinin	NP ↓, hPIV-2-F ↓, HA-NA ↓	hPIV-2	(73)
Quercetin	NP ↓	IAV	(70)
Cirsimaritin	M2 protein ↓	IAV	(68)
*G. thunbergii* extract	NP ↓, PA↓, M1 ↓, M2 ↓, PB 1 ↓, PB2 ↓, HA ↓, NA ↓	IAV, IBV	(52)
Isocorilagin (*Canarium album*)	NP ↓	IAV	(53)

* *only effects that are significantly different from the control are shown; ↓Parameter is significantly lower than the untreated virally- infected model; ↑Parameter is significantly higher than the untreated virally- infected model*.

*EGCG, Epigallocatechin gallate; F, RSV fusion protein; G. thunbergii, Geranii thunbergii; GSP, Grape seed proanthocyanidin; HA, hemagglutinin; HA-NA, hemagglutinin-neuraminidase; hPIV-2, Human parainfluenza virus type 2; hPIV-2-F, hPIV-2 fusion protein; IAV, influenza A virus; IBV, Influenza B virus; M1, matrix protein 1; M2, matrix protein 2; NP, nucleoprotein; PA, Polymerase acidic protein; PB1, Polymerase basic protein 1; PB2, Polymerase basic protein 2; RSV, Respiratory syncytial virus*.

Inhibition of viral polymerase activities have been observed in IAV-infected cells following treatments with iridoid glycosides (50). *G. thunbergii* extract was also reported to lower the levels of IAV polymerase transcript products including PA, PB1, and PB2 (52). Reduction in individual subunits of IAV polymerase would further impair the transcription and replication of IAV genome. It would also impair other specific functions of the individual polymerase subunits. For instance, PA-X (a transcript product of PA gene), is known to selectively shuts down the expression of host protein, enhance degradation of host mRNA and suppresses host antiviral responses (3). Therefore, reduction in the expression of PA proteins following treatments with *G. thunbergii* extract may likely impair IAV's ability to suppress host protein synthesis.

The evidence presented in this review shows that some PDFGS suppressed the expression of IAV M1 and M2 proteins in IAV infected cells (Table 3). The IAV-M gene encodes M1 and M2 protein which functions in the stabilization of RNP complex, viral replication and viral budding process (6, 97). M2 also function in the modulation of host ion channel activities, causing a pH dependent alteration in the permeability of ion channels (97). Inhibition of M2 ion channel is exploited in the therapeutic management of IAV infections and forms the mode of action of the commonly prescribed antiviral drug, *Amantadine* (52). The abilities of PDFGS such as curcumin, cirsimaritin and *G. thunbergii* to reduce the levels of IAV M2 (10, 52, 68) and *G. thunbergii*, EGCG, quercetin, isoquercetin and fisetin to reduce IAV M1 protein levels (29, 52) in IAV infected cells makes them potentially able to suppress IAV infectivity and proliferation.

#### PDFGS Interfered With RSV Lifecycle

PDFGS can interfere with different stages of RSV lifecycle Figure 6. For instance, treatments of RSV-exposed cells with *Z. officinale* extract, 18β-glycyrrhetinic acid (18β-GA) and blackcurrant extract inhibited the attachment of RSV unto host receptor proteins and consequently reduced RSV penetration into host cells (19, 20, 48). Quercetin was reported to interact irreversibly with RSV, as such, potentially limiting the interactions between RSV and host receptors (58). This explains the observed reduction in the infectivity of quercetin-pretreated RSV particles on tissue culture cell monolayers (TCCM) (58).

Treatment of RSV-infected cells with grape seed proanthocyanidin (GSP) suppressed the levels of RSV mRNA transcripts of fusion protein and nucleoprotein (59). Tangeretin was also reported to suppress the expression of different RSV mRNA transcripts including NS1, NS2, L, NP, M2-1, and M2-2 in RSV infected cells (69). Reduction of the mRNA synthesis is indicative of an impaired RSV transcription and replication functions. This assertion is further supported by the observed reduction in RSV replication in tangeretin treated, RSV infected A549 cells (69). Reduction in RSV replication and transcriptional activities would impair the synthesis of viral proteins as well as reduce the assembly of infective progeny RSV virions. This assertion is supported by the observed concomitant reduction of both mRNA and protein levels of RSV fusion protein following treatments with GSP (59). PDFGS such as 3,4-dicaffeoylquinic acid, 3,5-dicaffeoylquinic acid, fresh *Z. officinale* extracts, anthocyanin and 18β-GA were also shown to lower RSV titer in RSV-infected cells (19, 20, 48, 62).

#### Effect of PDFGS on Other Respiratory Viruses

PDFGS also interfered with the lifecycle of other respiratory viruses (Tables 2–4). Blackcurrant extract suppressed the attachment of AdV to cultured cell models hereby reducing AdV infectivity (48). Lectins such as concanavalin A, lens culinaris agglutinin and peanut agglutinin were observed to interact directly with host cell receptors hereby preventing hPIV-2 particles from accessing their cognate host receptor (73). Mannose specific plant lectins are equally capable of masking the cognate receptors of SARS coronaviruses on host cells hereby preventing a successful virus-host cell interaction (73).

**Table 4:** Effect of PDFGS on virus-host interaction & virus-cell entry function.

**Agent**	Targets/Effect^*^	**Viruses**	**References**
Curcumin	Antiviral agent-virus binding ↑	IAV	(10)
*R. acetosa* extract, EGCG	Antiviral agent-virus binding ↑ Viral penetration ↓ Viral attachment to host ↓	IAV	(55)
EBN	Antiviral Agent-viral interaction ↑, HA activity ↓	IAV	(57)
Quercetin	Antiviral agent-viral interaction ↑	RSV, hPIV-3	(58)
EGCG	Viral envelop damage ↑, Viral penetration to host ↓	IAV, IBV	(22)
Germacrone	Viral attachment to host cells↓	IAV, IBV	(60)
Quercetin	Viral entry ↓	IAV	(70)
Sulphated polysaccharide from *Aphanothece sacrum*	Viral attachment to host cells↓	IAV	(61)
Lectin-like compounds in Fruit-juice concentrate of Japanese plum (compounds)	HA activity ↓	IAV	(67)
Concanavalin A, lens culinaris agglutinin and peanut agglutinin	Actin disruption ↑, Viral attachment to host cells ↓, antiviral Agent-host interaction ↑	hPIV-2	(73)
Quercetin	Antiviral agent-virus HA2 binding ↑	IAV	(70)
Green tea, Guava tea	Viral heamagglutination ↓	IAV	(66)
*Z. officinale*	*Viral attachment↓, viral penetration ↓*	*RSV*	*(19)*
18β-GA	Viral attachment↓, viral penetration ↓	RSV	(20)
Caffeic, Chlorogenic, Gallic acid	Viral attachment to host cells↓	HCoV-NL63	(44)
Flavonoids(Isorhamnetin, quercetin, kaempferol)	Viral attachment to host cells↓	IAV	(51)
Isorhamnetin	HA activity ↓	IAV	(51)
Blackcurrant extract	Viral attachment to host cells ↓	AdV, IBV, IAV, RSV	(48)
*P. oleracea extract*	Antiviral agent-virus binding ↑	IAV	(25)
Adlay tea	Viral attachment to host cells ↓	IAV, IBV	(16)
HS	HA activity ↓	IAV	(45)
Tannins (gallic acid, pentagalloylglucose, tannic acid, EGCG)	HA activity ↓, antiviral agent-virus binding ↑, antiviral agent –host cell binding ↑	IAV	(41)
Polysaccharide extract from marine algae	HA activity ↓	IAV	(23)

* *only effects that are significantly different from the control are shown; ↓Parameter is significantly lower than the untreated virally- infected model; ↑Parameter is significantly higher than the untreated virally- infected model*.

*18β-GA, 18β-glycyrrhetinic acid; AdV, Adenovirus; EBN, Edible bird nest; EGCG, Epigallocatechin gallate; HA, hemagglutinin; HA2, fusion peptide; HCoV-NL63, Human coronavirus NL63; hPIV-2, Human parainfluenza virus type 2; hPIV-3, Human parainfluenza virus type 3; HS, Hibiscus sabdariffa L; IAV, influenza A virus; IBV, Influenza B virus; P. oleracea L, Portulaca oleracea L; R. acetosa, Rumex acetosa; RSV, Respiratory syncytial virus; Z. officinale, Zingiber officinale*.

Treatment of Rhesus Monkey Kidney Epithelial (LLC-MK2) cells with concanavalin A and lens culinaris agglutinin increased cytoskeletal disruption in hPIV infected cells (73). Disruption of cellular cytoskeleton can potentially interferes with hPIV-3 cellular transport, transcription and replication activities (73). Quercetin was also reported to bind irreversibly with hPIV-3 following a pretreatment of PIV with quercetin (58). This lowered the ability of hPIV-3 to infect tissue culture cell monolayers (58).

Phenolic constituents of Sambucus FormosanaNakai extract, including caffeic acid, chlorogenic acid, and gallic acid were shown to inhibit the attachment of HCoV-NL63 to LLC-MK2 cells (44). Caffeic acid possibly interacted with angiotensin-converting enzyme (ACE) and heparan sulfate on host cell surfaces hereby inhibiting the attachment of HCoV-NL63 to cellular receptors (44). Treatments of HCoV-NL63 exposed LLC-MK2 cells with caffeic acid, chlorogenic acid, and gallic acid was further observed to repress the release of progeny HCoV-NL63 virions (44).

Treatments of hPIV-2 infected cells with concanavalin A, lens culinaris agglutinin and peanut agglutinin significantly decreased the expression of hPIV-2 NP mRNA as well as lower the protein levels of hPIV-2 NP, hPIV-2 fusion protein (hPIV-2-F), and hPIV-2 hemagglutinin-neuraminidase (73). This could adversely affect viral entry, replication and release function hereby suppressing the proliferation of hPIV-2 particles (73). Treatments of RV1B infected cells with vitamin D (Vit D) was observed to inhibit the synthesis of viral RNA as well as suppress the release of progeny RV1B virions (72). The infectivity and replication of PIVs was also suppressed following treatment of infected cells with quercetin, hesperetin, and catechin (58).

#### Effect of PDFGS on Host Antiviral Immune Response

Cell membranes contain pattern recognition receptors (PPR) such as TLR, which senses for pathogenic associated molecular patterns (PAMP), such as viral nucleic acids, around the extracellular spaces (98). Endosomal and lysosomal membrane contains other PPR such as melanoma differentiation-associated protein 5 (MDA-5) and retinoic acid-inducible gene I (RIG-I), which senses for viral nucleic acids in cytoplasmic spaces (98). The PPR (TLR, MDA-5, and RIG-1) are activated following recognition and binding of viral nucleic acids (98). Activation of PPR leads to the activation of transcription factors like Nuclear factor kappa B (NF-κB), interferon regulatory factor (IRF), activator protein-1 (AP-1) which enhances the downstream expression of type 1 and type 3 interferons, proinflammatory cytokines and ISGs (3, 98).

Interferons functions to eradicate viruses by enhancing the expressions of antiviral ISGs such as Myxovirus Resistance Gene A (MxA) and Myxovirus Resistance Gene B (MxB), protein kinase R (PKR), 2′-5′ oligoadenylate synthetase (**OAS**), ribonuclease L (RNase L) and IFN-induced transmembrane (IFITM) through a signal transducer and activator of transcription (STAT) 1/2, Janus kinase 1 (JAK1) and non-receptor tyrosine-protein kinase (TYK2)-mediated signaling (3). In humans, MxA can inhibit viral transcription by binding to IAV NP (3). IFITM, largely localized on cell membranes, can prevent viral infection by interfering with virus-host cell interaction, attachment and endocytosis (3). Activated OAS enhances the degradation of viral single-stranded RNA (ssRNA) as such preventing viral replication and proliferation as well as stimulating further RIG-1 mediated antiviral responses (3). Activated PKR inhibits protein synthesis in viral infected cells (3). Detection of viral PAMP also causes the activation of NLR family pyrin domain containing 3 (NLRP3) inflammasome complexes, a component of the innate immune response. This stimulates the expression of proinflammatory interleukin 1 β (IL-1β) and interleukin 18 (IL-18) and pyroptosis in a caspase 1 dependent processes (3, 99).

The ability of viruses to infect and propagate in host environment depends on their ability to evade host immune systems. Viral proteins such as NS1 of IAV interact with RIG-1 to inhibit its downstream activities (98). Virus-induced shutdown of protein synthesis through the activities of viral PA-X, NS1, and NS2 results in overall downregulation of proteins involved in host's antiviral immune responses such as reduced RIG-1, interferon regulatory factor 3 (IRF3), NFkB, AP-1, PKR, OAS, NLRP3 inflammasome, and poly adenine binding protein II (PABPII) activation (3). NS1 of IAV can prevent Tripartite Motif Containing 25 (TRIM25) induced inhibition of IAV RNP (98).

This review revealed that PDFGS such as Vit D, acteoside, *Adansonia digitata (A. digitata)* leaves extract, 18β GA, and curcumin are able to boost host antiviral immune responses by upregulating various endogenous antiviral mediators (Table 5). VIt D was observed to stimulate the production of antiviral interferon-stimulated genes (ISGs) such as viperin and MxA, against RV1B in *in vitro* studies (72). Vit D also up-regulated the expression of cathelicidin, an endogenous antimicrobial peptide that was also shown to be virucidal against RV1B (72). Curcumin enhanced the expression of interferon β (IFN-β) against IAV in cultured cells (10). Extract from *A. digitata* leaves was observed to increase the expression of IL-8 in RV1A infected lung epithelial cells (64). Acteoside activated T-box expressed in T cells (T-bet) in immune cells with the resultant upregulation of interferon γ (IFN-γ) expression (65). Upregulation of these endogenous immune mediators can facilitate virus clearance in infected cells.

**Table 5:** Effect of PDFGS on host antiviral defenses.

**Agent**	Biomarker/Effect^*^	**Viruses**	**References**
Vit D	Viperin ↑, MxA ↑, Cathelicidin ↑	RV1B	(72)
Acteoside	IFN-γ ↑, T-bet ↑	IAV	(65)
*A. digitata* leaves extract	IL-8 ↑	RV 1A	(64)
18β-GA	Glycyrrhizin and IFN-β ↑	RSV	(20)
Curcumin	IFN-β ↑	IAV	(10)

* *only effects that are significantly different from the control are shown; ↓parameter is significantly lower than the untreated viral infected model; ↑parameter is significantly higher than the untreated viral infected model*.

*18β-GA, 18β-glycyrrhetinic acid; A. digitata, Adansonia digitata; IAV, influenza A virus; IFN-β, Interferon β; IFN-γ, Interferon γ; IL-8, interleukin 8; MxA, Myxovirus Resistance Gene A; RV1B, rhinovirus 1A; RV1B, rhinovirus 1B; RSV, Respiratory syncytial virus; T-bet, T-box expressed in T cells; Vit D, Vitamin D*.

The upregulation and activation of signaling cascades that generate endogenous antiviral molecules like interferons (IFN) and ISGs may reverse virus-induced suppression of host immune response and so facilitate the clearance of virus infected cells as well as reduce virus titer. Exogenous interferon α (IFN-α) is commonly administered with ribavirin for the treatment of different viral diseases (100, 101). Evidence from this review highlights the prospects of 18β-GA, curcumin, Vit D and acteoside in boosting cellular immunity against respiratory viruses, through the upregulation of interferon expression.

#### Potential Mechanism of Respiratory Virus-Induced Cytotoxicity

Ordinarily, the TLR and RIG-1 mediated expression of immune and inflammatory mediators is a protective response to viral infection (3, 98). Such response is beneficial only when it is localized to the region of infection. It can however trigger a potentially life threatening “cytokine storm” if it becomes systemic (102). As such, Innate immune response is usually under strict regulation so as to ensure that cells produce sufficient but not excessive immune responses to antigens (98). Virus infections are often known to cause hyper active inflammatory responses (103, 104). This may be contributed in part by a dysfunctional regulation of components of cellular immunity (105) such as a dysregulated post-translational modifications of some key actuators of innate immunity (98). Virus-induced inflammations could also lead to ER stress with further generation of inflammatory mediators (106). The ability of some respiratory viruses to interfere with critical processes such as autophagy and apoptosis (6, 107) makes them potentially able to cause dysregulation of different cellular processes including immune responses.

**Figure 7:**
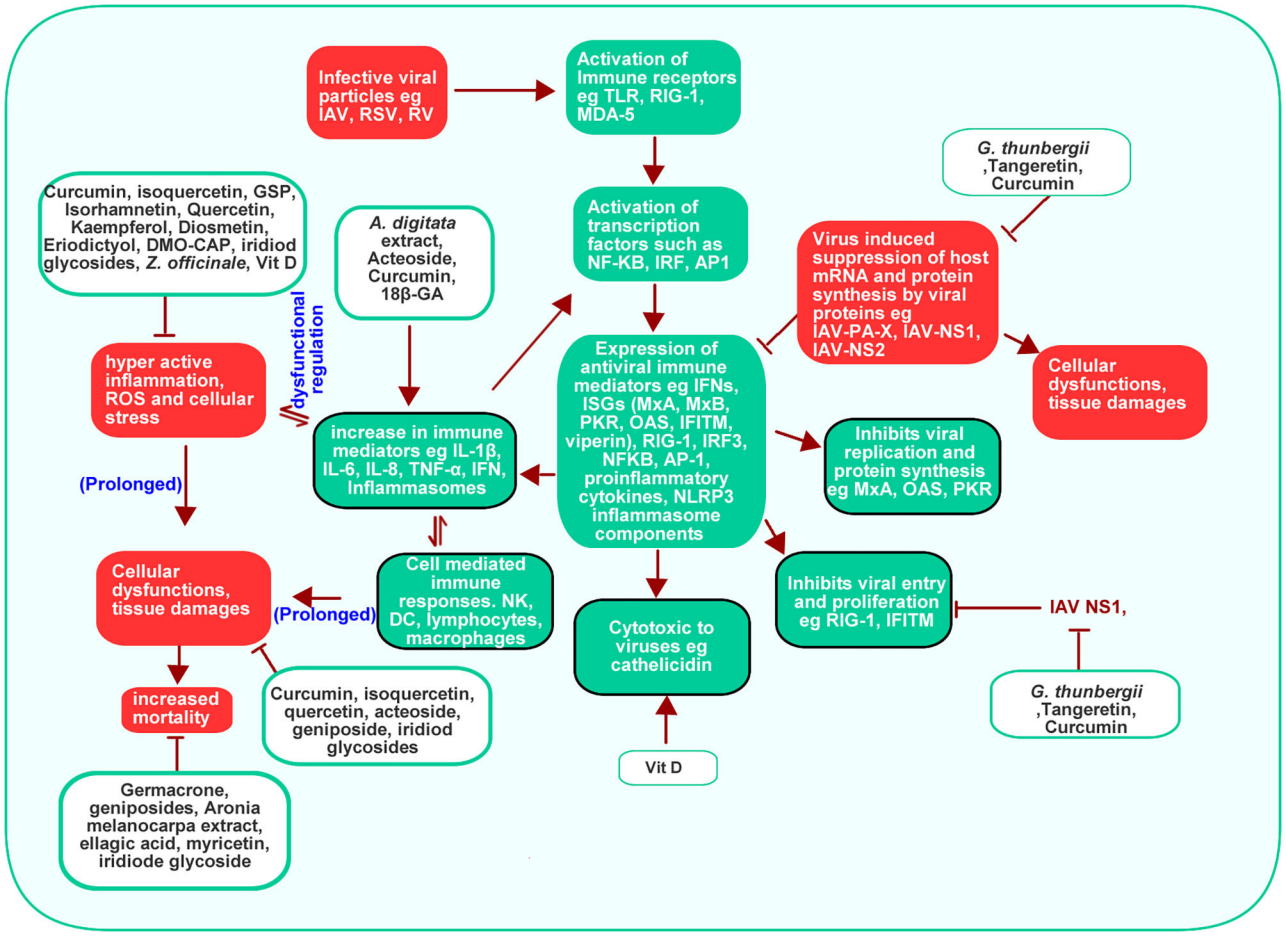
Hypothetical mechanism of respiratory viruses-induced cellular damages. Activation of TLR receptor by viral RNA leads to the generation of localized inflammation at sites of infection. This inflammation may progress into hyper-inflammation if the virus succeeds in evading host antiviral defenses or if viral infection is prolonged. This consequently leads to the distortion of cellular homeostasis including the upregulation of wnt/b-catenin, AKT, and MAPK signaling. Persistent activation of these signaling cascade may trigger endoplasmic reticulum stress resulting in further oxidative stress and inflammation which eventually leads to tissue damages. AKT, Protein kinase B; MAPK, Mitogen-activated protein kinase; NF-κB, Nuclear factor-κB; ROS, Reactive oxygen species; TLR, Toll-like receptor.

Persistent inflammatory stress signals in cellular environments can lead to alteration of cellular homoeostasis which could potentially lead to increased cellular stress and various pathological consequences (108, 109). For instance, inflammatory conditions are known to upregulate the wnt/β-catenin pathway (110, 111) with resultant enhancement of apoptosis (112). Increased levels of ROS have been observed in some respiratory virus infection (10). ROS have been reported to upregulate phosphatidylinositol-3-kinase (PI3K)/protein kinase B (AKT) signaling in cancer cells (111). MAPK/p38 signaling is activated under cellular stress and can enhance the generation of inflammatory cytokines and apoptosis (113–115). The result presented in this review showed that respiratory viruses promoted the activation of the AKT and MAPK/p38, Wnt3a/catenin signaling cascade (10, 86). Increased activity of these signaling pathways can cause endoplasmic reticulum stress and oxidative stress leading to further inflammatory response, autophagy and apoptosis as such causing further cellular damages (106, 116, 117). A hypothetical virus-mediated processes that builds up to tissue damages is shown in Figure 7.

Increased loss of weight, lung lesions, lung index are some of the respiratory virus derived toxicity that can be promoted by sustained cellular stress conditions (118–120). For instance, COPD, which is characterized by a gradual destruction of the lung parenchyma and alveolar structures, has been associated with increased inflammatory and oxidative stress-induced damage (120). Pulmonary and systemic inflammations induced by severe acute respiratory syndrome coronavirus 2 (SARS-CoV-2) infection can cause multi-organ dysfunction and consequently increases the risk of mortality in some patients (121).

#### PDFGS Suppressed Virus-Induced Cellular Damages

**Figure 8:**
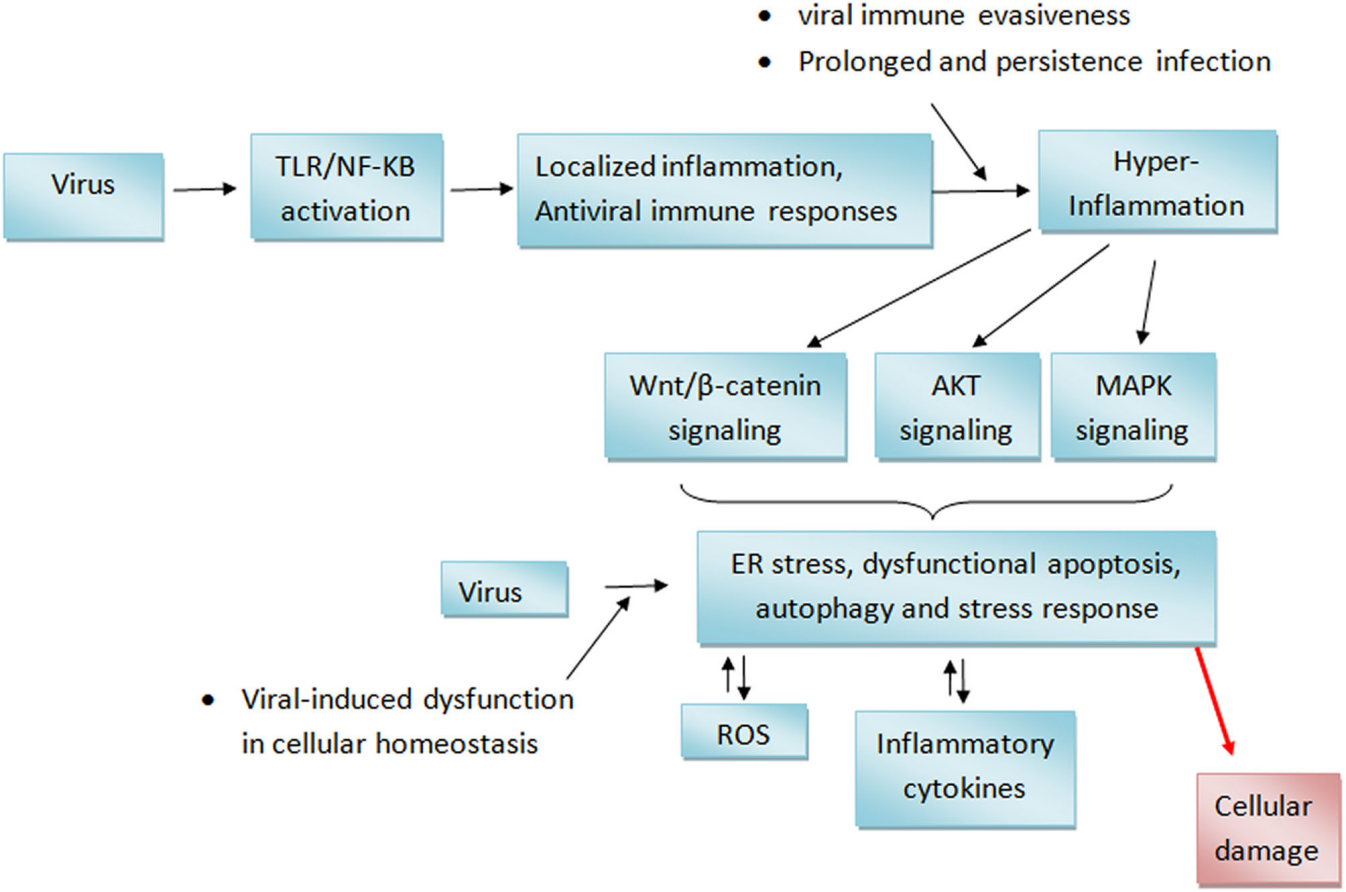
Effect of PDFGS on host antiviral immune responses. Membrane resident immune receptors such as TLR senses viral RNA. This stimulates the expression of transcription factors which subsequently enhance the production of antiviral peptides and other immune mediators. Viruses induced suppression of host protein synthesis can downregulate the synthesis of antiviral immune mediator and can potentially lead to a dysfuntional cellular homeostasis. This could lead to inflammation and consequently, cause damages to tissues. PDFGS suppresses viral proteins, enhances the exepression of antiviral proteins as well as suppress virus-induced inflammations. 18β-GA, 18β-glycyrrhetinic acid; *A. digitata, Adansonia digitata*; AP-1, Activator protein-1; DC, Dendritic cells; DMO-CAP, 6-demethoxy-4′-O-methylcapillarisin; *G. thunbergii, Geranii thunbergii;* GSP, Grape seed proanthocyanidin; IAV, Influenza A virus; IAV-NS1, IAV non-structural protein 1; IAV-NS2, IAV non-structural protein 2; IAV-PA-X, transcript product of IAV polymerase; IFITM, IFN-induced transmembrane; IFN, Interferons; IL-1β, Interleukin 1β; IL-6, Interleukin 6; IL-8, Interleukin 8; IRF, Interferon regulatory factor; IRF3, Interferon regulatory factor 3; MDA-5, Melanoma differentiation-associated protein 5; MxA, Myxovirus Resistance Gene A; MxB, Myxovirus Resistance Gene B; NF-κB, Nuclear factor kappa B; NK, Natural Killer cells; OAS, 2'-5' oligoadenylate synthetase; PKR, Protein kinase R; RIG-1, Retinoic acid-inducible gene I; RNase L, Ribonuclease L; ROS, Reactive oxygen species; RSV, Respiratory syncytial virus; RV, Rhinovirus; TLR, Toll-like receptor; TNF-α, Tumor necrosis factor-α; Vit D, Vitamin D; *Z. officinale, Zingiber officinale*.

The data presented in this review showed that certain PDFGS including geniposide, acteoside, curcumin, and flavonoids can reverse respiratory virus-induced cytotoxicity and so increase the survival of virus-infected animal models (Table 6). These PDFGS reduces respiratory virus-induced pathologies by suppressing respiratory virus-induced expression of pro-inflammatory mediators [such as tumor necrosis factor-α (TNF-α), interleukin 6 (IL-6), IL-8, IFN-γ, inducible nitric oxide synthase (iNOS), and regulated on activation normal T cell expressed and secreted (RANTES) (Figure 8)]. DMO-CAP and certain flavonoids are able to suppress virus-induced generation of reactive oxygen species (ROS) and virus-induced depletion of cellular antioxidant capacities including Nrf2, heme oxygenase-1 and glutathione/glutathione disulfide (42, 51). PDFGS such as GSP, acteoside, and curcumin inhibited virus-induced activation of potentially cytotoxic signaling cascades such as extracellular signal-regulated kinase (ERK), c-Jun N-terminal kinase (JNK) and P38 mitogen-activated protein kinase (MAPK) signaling; TLR, myeloid differentiation primary response 88 (MyD88), TIR-domain-containing adapter-inducing interferon-β (TRIF), and Tumor necrosis factor receptor (TNFR)-associated factor 6 (TRAF6) signaling; IkB kinase (IKK)/NF-kB signaling; Wnt family member 3a (wnt3a)/β-catenin signaling and autophagic signaling (10, 51, 59, 86). Curcumin and GSP also reduced the production of mucin proteins, pulmonary matrix metalloproteinase (MMP) and collagen (10, 59, 86).

**Table 6:** Effects of PDFGS on virus-induced cytotoxicity.

**Agent**	Biomarker/Effect^*^	**Viruses**	**References**
Curcumin	TNF-α ↓, IL-6 ↓, IL-1β ↓, I collagen ↓, and MMP-2 mRNA ↓ Organ to body weight ratio ↓, WNT3A ↓, B-catenin ↓, TCF4, Cyclin D1 ↓, c-MYC ↓	IAV	(86)
Curcumin	TNF- α ↓, IFN- α ↓, IL-6 ↓, iKB ↓, NFkB-p65 ↓, neutrophils ↓, lymphocytes ↓, macrophages ↓	IAV	(43)
Curcumin	Pulmonary cytokines (TNF-α, IL-1β, IL-6, and IL-8) ↓ Pulmonary MMP (MMP-2 and MMP-9) ↓, ROS ↓, GSH/GSSG ↑, GSTA3 ↑, Nrf2 ↑, HO-1 ↑, TLR2/4/7-MyD88/TRIF_TRAF6 signaling ↓, Akt signaling ↓, p38/MAPK signaling ↓and NFkB signaling ↓ pathways, Survival rate ↑, lung index ↓	IAV	(10)
Isoquercetin	IFN-γ ↓, iNOS↓, RANTES↓	IAV, IBV	(29)
GSP	iKB/NFKBp65↓	RSV	(59)
Isoquercetin	Viral bronchitis↓, Epithelial damage ↓	IAV, IBV	(29)
Quercetin	Viral induced hemolysis ↓	IAV	(70)
Cirsimaritin	IL-10 ↓, TNF- α ↓, NFkB ↓, IL-8 ↓, IL-1β ↓, JNK/MAPK/P38 ↓, COX2 ↓	IAV	(68)
Flavonoids (Isorhamnetin, Quercetin, Kaempferol, Diosmetin, Eriodictyol)	ROS ↓, Cellular damage ↓, Survival rate ↑, weight loss ↓, RBC hemolysis (isorhamnetin) ↓, p-ERK ↓, Autophagy (LC3B) ↓.	IAV	(51)
GSP	ERK/JNK/p38 ↓, AP1 (cjos and cfos) ↓, mucin proteins (MUC1, MUC2, MUC5AC, MUC5B, MUC8)↓	RSV	(59)
Germacrone	Mortality ↓	IAV, IBV	(60)
Acteoside	Lung lesion ↓, lung index ↓, p-ERK/ERK ↑	IAV	(65)
Geniposide	Weight loss ↓, Mortality ↓, lung lesion ↓, alveolar wall change ↓, alveolar hemorrhage ↓, neutrophils-infiltration in lung tissue ↓, cell damage ↓ IL-4 ↑, IL-10 ↑, TNF- α ↓, IFN-γ↓, IL-6↓	IAV	(71)
DMO-CAP	Antioxidant capacity (HO-1, Nrf2, MAPK pathway) ↑	IAV	(42)
*A. melanocarpa* extract, ellagic acid, myricetin	Survival rate ↑, weight loss ↓,	IAV	(46)
Iridoid glycosides	Pulmonary index ↓, weight loss ↓, Survival rate ↑, PACT ↓, PKR↓, IFN-β ↓, PACT/PKR/elF2α signaling↓	IAV	(50)
*Z. officinale*	TNF-α ↓	RSV	(19)
Vit D	IL-6 ↓, IL-8↓	RV1B	(72)
Vit D	NFKB ↓, iKB-α ↑ IFN- β ↓, CXCL10 ↓, STAT1 ↓, MxA ↓, ISG15 ↓	RSV	(49)

* *only effects that are significantly different from the control are shown; ↓Parameter is significantly lower than the untreated virally- infected model; ↑Parameter is significantly higher than the untreated virally- infected model*.

*A. melanocarpa, Aronia melanocarpa; Akt, Protein kinase B; AP1, Activator protein-1; COX2, cyclooxygenase-2; CXCL10, C-X-C motif chemokine ligand 10; DMO-CAP, 6-demethoxy-4′-O-methylcapillarisin; elF2α, Eukaryotic Initiation Factor 2; ERK, Extracellular signal-regulated kinase; GSH, Glutathione; GSP, grape seed proanthocyanidin; GSSG, Glutathione disulfide; GSTA3, Glutathione S-transferase A3; HO-1, Heme Oxygenase; IAV, influenza A virus; IBV, Influenza B virus; IFN-β, Interferon β; IFN-β, Interferon β; IFN-γ, Interferon γ; IL-1, interleukin 1β; IL-10, interleukin 10; IL-6, interleukin 6; IL-8, interleukin 8; iNOS, Inducible nitric oxide synthase; ISG15, interferon-stimulated gene 15; JNK, c-Jun N-terminal kinase; MAPK, Mitogen-activated protein kinase; MMP, Matrix metallopeptidase; MMP-2, Matrix metallopeptidase 2; MMP-9, Matrix metallopeptidase 9; MUC1, Mucin 1; MUC2, Mucin 2; MUC5AC, Mucin 5AC; MUC5B, Mucin 5B; MUC8, Mucin 8; MxA:Myxovirus Resistance Gene A (MxA); MyD88, Myeloid differentiation factor 88; NF-κB, Nuclear factor-κB; Nrf2, Nuclear factor erythroid 2–related factor 2; p-ERK, Phosphorylated Extracellular signal-regulated kinase; PKR, Protein kinase R; RANTES, Regulated upon Activation, Normal T Cell Expressed and Presumably Secreted; RBC, red blood cell; ROS, reactive oxygen species; RSV, Respiratory syncytial virus; RV1B, rhinovirus 1B; STAT1, signal transducer and activator of transcription; TCF4, Transcription factor 4; TLR 2/4/7, Toll like receptors 2/4/7; TNF-α, Tumor necrosis factor-α; TRAF6, Tumor necrosis factor receptor (TNFR)-associated factor 6; TRIF, TIR-domain-containing adapter-inducing interferon-β; Vit D, Vitamin D; wnt3a, Wnt family member 3a; Z. officinale, Zingiber officinale*.

Acteoside stimulated an increased phosphorylation of the pro-survival ERK in mice infected with IAV (65). Some PDFGS including Iridoid glycosides, certain flavonoids, curcumin were reported to reduce virus-induced weight loss, lung lesion, lung index, alveolar damages, hemolysis, cellular damages as well as improved survival rates of virus-infected animals (10, 50, 51, 70, 71). Curcumin and geniposide also suppressed virus-induced increase in the numbers of neutrophils, lymphocytes, and macrophages (43, 71).

The evidence collated in this review showed that PDFGS such as curcumin, flavonoids, ginger, calcitriol, iridoid glycosides, and geniposide are able to suppress respiratory virus-induced hyperactive immune and inflammatory responses together with their potential cytotoxic effects. Corticosteroids are widely used as adjuvant therapy in managing respiratory virus infection because of their anti-inflammatory and immunomodulatory properties (122, 123). The Suppression of AKT, wnt3a/β-catenin and TLR signaling by curcumin (10, 86); MAPK signaling by curcumin, acteoside and flavonoids (10, 51, 65); IFN expression by curcumin, calcitriol, geniposide, and isoquercetin (29, 60, 71, 72) in respiratory virus exposed organisms have the potential to suppress inflammation and its associated cellular consequences. Enhancement of cellular antioxidant capacity by some of the PDFGS such as curcumin, DMO-CAP and different flavonoids (10, 42, 51), would reduce virus-induced oxidative stress and their potential cytotoxic effects. The cumulative effect of these PDFGS is the amelioration of viral induced cellular damages. This is evident from the observed reduction in lung lesions, pulmonary index, weight loss and lungs index as well as the increased survival rates of virus-infected organisms treated with some of the PDFGS highlighted in this review (10, 29, 50, 51, 71).

#### PDFGS Ameliorated Respiratory Viral Infections in Mice Experiments

Data obtained from *in vivo* studies indicates that PDFGS equally exerted beneficial effects against mice models of IAV infections (Table 7). Treatments of mice infected with pandemic influenza A/Jiangsu/i/09 (H1N1) virus, with 20 mg/kg of geniposide administered intraperitoneally for 14 days, ameliorated IAV-induced weight loss, reduced IAV-induced lung lesions, improved mice survival rates and decreased viral proliferation in infected mice models (71). Geniposide also reduced the expression of IAV NP in IAV-infected mice as well as reduced the levels of inflammatory mediators such as TNF-α, IL-6, and IFN-y (71). Geniposide-mediated suppression of caspase 3 activation in IAV-infected epithelial cells, suppressed the export of IAV RNP complexes to the cell membrane (71). This can potentially inhibit the packaging of infectious progeny virions (71).

**Table 7:** Summary table of included *in vivo* studies.

**Antiviral PDFGS**	**Sources**	**Susceptible Virus/host organism**	**Intervention**	**Antiviral effect**	**Effective antiviral concentration**	**Antiviral targets**	**References**
Isoquercetin		mouse-adapted IAV(H1N1)/mice	5, 10 mg/kg/day for 8 days, intraperitoneally	Reduction in lung viral titer (19-folds)	10 mg/kg	Viral titer reduction, virus-induced cytotoxicity	(10)
Curcumin		IAV(H1N1)/mice	50, 150 mg/kg, twice daily for 6 days, orally	Improved Survival rate (70%)	150 mg/kg	Viral titer reduction, virus-induced cytotoxicity	(29)
Curcumin		IAV (H1N1)/mice	50–400 mg/kg administered daily intraperitoneally for 4 days	Reduction immune cell count (75%) Reduction in lymphocytes (66%)	400 mg/kg	Viral-induced cytotoxicity	(43)
Geniposide	*Gardenia jasminoides* fruit	IAV (H1N1)/mice	5,10,20 mg/kg, intraperitoneally for 14 days	Lung Viral titer reduction (36%)	20 mg/kg	Viral replication, Virus-induced cytotoxicity	(71)
Acteoside (phenylpropanoid glycoside)	Kuding Tea	IAV (H1N1)/Mice	80 mg/kg administered intraperitoneally	>20-folds increase in IFN-γ production	80 mg/kg	Host defense	(65)
Curcumin	*Curcuma longa* L	IAV (H1N1)/Mice	100 mg/kg/day for 5 days orally	Significant reduction in the expression of proinflammatory genes	100 mg/kg	Virus-induced cytotoxicity	(86)
Germacrone	*Rhizoma Curcuma*	IAV (H1N1)/Mice	50–100 mg/kg/day administered intraperitoneally for 5 days	Mortality reduction (50%)	100 mg/kg	Virus-induced cytotoxicity	(60)
Isorhamnetin, Quercetin, Kaempferol, Diosmetin, Eriodictyol		IAV(H1N1)/mice	1 mg/kg/day administered intranasally for 5 days	Lung titer reduction (2-folds), Improved survival rate (70–80%)	1 mg/kg	Virus-induced cytotoxicity, Viral proliferation	(51)
*A. melanocarpa* extract, Ellagic acid, myricetin	Black chokeberry	IAV(H1N1)/mice	1 mg/kg administered twice daily for 5 days. *A. melanocarpa* extract was administered orally, while Ellagic acid and myricetin were given intraperitoneally	Lung titer reduction (> 50%), Reduction in viral replication (15–30%)	1 mg/kg	Virus-induced cytotoxicty, viral replication	(46)
*Hibiscus sabdariffa* extract	*Hibiscus sabdariffa*	IAV (H1N1)/mice	8 mg/mice of *Hibiscus sabdariffa* tea extract was administered orally twice daily for 14 days.	No significant difference in IAV induced mortality	–	–	(45)
Iridoid glycosides	*Fructus Gardeniae*	IAV (H1N1)/mice	5,10,20 mg/kg orally for 4 days	Mortality reduction (52%)	20 mg/kg	Viral replication, Virus- induced cytotoxicty	(50)

*IAV, influenza A virus; IBV, Influenza B virus; IC50, Half maximal inhibitory concentration; LLC-MK2, Rhesus Monkey Kidney Epithelial Cells; MDCK, Madin-Darby Canine Kidney; MHV, Murine hepatitis Virus; MIC50, Minimum Inhibitory Concentration required to inhibit the growth of 50% of organisms; R. acetosa, Rumex acetosa; RSV, Respiratory syncytial virus; TD50, Median toxic dose; Vit D, Vitamin D; Z. officinale, Zingiber officinale*.

Treatment of recombinant IAV H1N1/PR8 expressing green fluorescent protein (rPR8-GFP)—infected mice, with 1 mg/kg of either *A. melanocarpa* extract (orally), ellagic acid [intraperitoneal (i.p)] or myricetin (i.p), for 3 days, significantly lowered IAV lung titer and viral replication as well as enhanced survival in IAV infected mice (46). The anti-IAV activity of *A. melanocarpa* was attributed to its rich polyphenol contents (46). Significant reduction in lung IAV titer, decreased weight loss, and increased survival rates were observed following intranasal administration of isorhamnetin (1 mg/kg/day for 5 days) to IAV (A/Puerto Rico/8/34 H1N1) infected mice (51). A significant reduction in lung virus titer (19-folds) as well as reduced expression of lung IFN-y, iNOS, and RANTES was observed following the treatment of IAV-infected mice models with isoquercetin (10 mg/kg/day, intraperitoneally, 8 days) (29). Intraperitoneal administration of acteoside (80 mg/kg) to mice infected with mice-adapted IAV FM1 strain (A/FM/1/47 H1N1) significantly reduced the severity of IAV-induced damages to the lung tissues of the IAV infected mice (65). The protective effect of acteoside was attributed to a possible enhancement of antiviral immune response without its associated inflammatory damages (65).

Treatment of mice models of IAV (Influenza A/Puerto Rico/8/34 H1N1) infection with curcumin (100 mg/kg, taken orally for 5 days) led to a significant reduction in the cardiac myocarditis, weight loss and heart inflammations, induced by IAV (86). Curcumin treatment also reduced IAV-induced increase in the expression of TNF, IL-1B, IL-6, and MMP-2 mRNA as well as suppressed IAV-induced increase in wnt/β-catenin signaling in IAV infected mice (86). Oral administration of curcumin (50, 150 mg/kg) was also reported to inhibit IAV replication, reduce lung viral titer and suppress virus-induced injuries in IAV infected mice (10). In a similar way, I.p injection of curcumin (100, 200, and 400 mg/kg for 4 days) to Influenza A/Puerto Rico/8/34 (H1N1; PR/8) virus-infected mice model significantly reduced IAV-induced increase in the numbers of macrophages, total immune cells, neutrophils and Lymphocytes (43). It also suppressed IAV-induced increase in the expression of TNF, IFN-alpha and IL-6 in bronchoalveolar lavage fliud of mice infected with IAV (43). The evidence reported by Liu et al. (86) suggests that the inhibition of wnt/β-catenin signaling by curcumin may be responsible for the suppression of IAV-induced cardiac myocarditis, following treatments of IAV-infected mice with curcumin. The ability of curcumin to suppress IAV NS1 gene expression in mice (86), also implies that curcumin can potentially inhibit the proliferation of IAV *in vivo*.

Intravenous injection of germacrone (100 mg/kg/day for 5 days) to IAV (influenza A/Puerto Rico/8/34 H1N1) exposed mice, significantly inhibited IAV *in vivo* and enhanced survival of IAV infected mice (60). The evidence presented by Liao et al. (60), further suggests that an additive anti-IAV benefit could be derived when germacrone is used in combination with oseltamivir. Treatment of virus (influenza virus strain A/FM/1/47) infected mice with iridiod glycosides, administered orally (5–20 mg/kg) for 5 day, protected infected mice from IAV-induced lung injury (50). Furthermore, a 52% reduction in mortality was observed following treatments of IAV-infected mice models with 20 mg/kg of iridiod glycosides. The observed antiviral effect was attributed to a possible PACT-dependent inhibition of IAV replication (50).

HS, despite having an *in vitro* anti-IAV activity, *in vivo* studies showed that the aqueous extract of HS (8 mg), neither improved survival nor reduce virus titer in PR8 infected mice (45). Furthermore, the evidence they presented suggested that the observed *in vitro* anti-IAV activity of HS was due to the acidic nature of HS extract (45). In a separate experiment where they vaccinated mice with IAV inactivated by acidic HS, they observed an improvement in survival rate following subsequent IAV challenge. They however argued that the acidic nature of HS extract makes it unsuitable for vaccine development due to the possible alteration of IAV HA conformation and antigenic characteristics (45).

Cumulatively, the evidence obtained from the animal studies, as presented in this review, shows some reasonable agreement with those obtained from *in vitro* studies.

#### Safety, Bioavailability, and Clinical Translation of PDFGS Against Respiratory Viral Infections

The evidence presented in this review indicates that some PDFGS are able to suppress viral release, viral titer, viral cytopathic effects, and plague formation at doses that are not cytotoxic to the models considered. Majority of the PDFGS exerted their antiviral effects at doses that ranged between 0.1 and 600 μg/ml or 0.1–200 μM (Table 1). In almost all the studies, the half maximal inhibitory concentration (IC50 or EC50) was lower than the 50% cytotoxic doses (TC50 or CC50) by a reasonable margin. Some of the PDFGS had IC50 that was within the range reported for antiviral drugs like ribavirin (2.4–22 μM), oseltamivir (0.01–0.5 μM), and amantadine (0.51–100 μM) (22, 29, 60, 61, 68).

The potency of an agent depends on its ability to attain the therapeutic dose at cellular sites of action. The *in vitro* nature of most of the included studies made attaining these effective antiviral doses possible. In reality, several factors such as intestinal metabolism, intestinal absorption, liver metabolism, gut microbiota metabolism, renal clearance and organ selective uptake affects the concentration of a particular agents at target cellular environments (124–129). Evidence obtained from some other studies suggests that the concentrations at which some of the PDFGS (highlighted in this review) elicited their antiviral effect might not be attainable from normal dietary intake of these PDFGS. For instance Sablok et al. (130) and Mark et al. (131) reported a blood Vit D level of <100 nmol in humans whereas 1,000 nmol Vit D was reported to suppress viral release by 60% (72). Intake of 8 grams of curcumin in human subject gave a peak plasma level of 1.77 μM (0.683 μg/ml equivalent) (126), which is less than the antiviral dose of 21.36 μg/ml reported by Dai (10). A peak plasma level (Cmax) of 385 nmol chlorogenic acid was attained in human subjects after ingestion of 412 μM of chlorogenic acid (125). Intake of pomegranate juice containing 318 mg of the elagitannin, punicalagins, gave a Cmax of 60 nmol/L ellagic acid after an hour (125). These values are less than the antiviral-effective doses of 2.78–43.45 μM and 3.64–119 μM reported for chlorogenic acids and tannin derivatives (41, 44, 62), respectively.

The bioavailability of many PDFGS following oral intake are largely limited by poor intestinal absorption. Fernandez-Arroyo et al. (132) reported that only about 1% of the ingested polyphenol is absorbed after an intake of polyphenol-rich extract of HS. The bioavailability of curcumin is further limited by its binding to enterocyte proteins (126). Since bioavailability of phytochemicals such as polyphenols are dose dependent, an unusually high amount of the plant agent needs to be consumed in order to attain plasma levels that are comparable to doses reported to elicit antiviral effect. Alternatively, the bioactive components may be enriched from the parent materials and consumed as supplements. Fernandez-Arroyo et al. (132) reported a plasma level of 5 μM (1.51 mg/ml quercetin equivalent) flavonols within 2 h of ingesting of 1,200 mg/kg of HS polyphenol extract by human subjects. Human subjects that ingested grape seed juice containing 10 mg of quercetin had Cmax of 16 μM (133) which falls within the antiviral-active concentrations of 5.4–17.3 μM as reported by Kim et al. (22) and Xu et al. (69). Potential biotransformation of the PDFGS under cellular conditions can affect the antiviral efficacy of PDFGS in human subjects. An instance is the recently reported association of the *in vitro* antiviral effects of HS extracts to their acidic properties (45). The buffering capacity of human cellular environment could potentially neutralize the HS acidity and so attenuate the observed *in vitro* antiviral effects of HS.

With the exceptions of a few studies, most of the other *in vivo* evidences presented in this review administered PDFGS intraperitoneally. These non-oral routes of delivery, coupled to the relatively high doses of the PDFGS administered to model animals, further underscores the challenge inherent in attaining therapeutic concentrations of the PDFSG at target sites of action. Derksen et al. (55), suggested formulating the PDFGS into chewing gums, inhaling devices and as lozenges as a way to increased their bioavailability at target respiratory tract sites. Dayem et al. (51), was able to achieve antiviral efficacy at lower concentration (1 mg/kg) by administering the PDFGS intranasally, to IAV-infected mice.

### Categories and Food Sources of the Antiviral PDFGS

This review collated evidences on the potential roles of dietary constituents in the management of respiratory viral diseases. This review identified antiviral PDFGS which falls into the categories of flavonoids, tannins, phenolic acids and other polyphenols, polysaccharides, lectins, Vit D, and plant glycosides.

#### Flavonoids

Flavonoids are ubiquitous polyphenols commonly found in fruits and vegetables. Evidence presented in this study indicates that different flavonoids including myricetin, isorhamnetin, quercetin, kaempferol, diosmetin, eriodictyol, quercetin 3-rhamnoside, procyanidin B2 digallate, grape seed proanthocyanidin, isoquercetin, fisetin, glycosyl hesperidin, tangeretin, and 6-demethoxy-4′-O-methylcapillarisin, interfered with different stages of respiratory virus's lifecycle and suppressed virus-induced pathologies. Myricetin, quercetin, isoquercetin, kaempferol are constituents of tea, onions, red wine, grapes, blackcurrants, and many edible berries (29, 134–136). Isorhamnetin is contained in onions (137). Diosmetin is abundant in oregano spice and in citrus fruits (138). Eriodictyol are constituent of wine, tea, and coffee (139). Cirsimaritin is present in *Ocimum gratissimum* (140), *Rosmarinus officinalis* leaves [used as spices and flavoring agent (141)] and in *Artemisia scoparia* [a herbal and culinary plant (68, 142)]. Catechin is abundant in cocoa and tea plant (124)]. Fisetin is abundant in fruits (strawberries, apples, persimmons, mangoes, kiwis, and grapes etc.), vegetables (tomatoes, onions, and cucumbers etc.), nuts, and wine (143, 144). Hesperitin, hesperidin and tangeretin are abundant in citrus fruits (145, 146). Glucosyl hesperidin is a synthetic and soluble derivative of hesperidin and possess similar metabolic and pharmacokinetic properties as the natural hesperidin (147). Powdered grape seed (containing procyanidins, gallic acid, catechin, epicatechin etc.) or proanthocyanidin rich extracts of grape seed are often consumed as supplement (148). Oligomeric proanthocyanidin are also present in red apple, cocoa, cinnamon, grapes etc. (149). DMO-CAP is a prenylated flavonoids and a constituent of *artemisia spp* such as *Artemisia dracunculus L* (42). Essential oils from *Artemisia dracunculus L*. are used as food spices and in herbal preparations (150).

#### Phenolic Acids

Phenolic acids, comprising the hydroxybenzoic derivatives such as gallic, vanillic, and syringic acids and their hydroxycinnamic acids derivatives such as caffeic, ferulic, sinapic, and p-coumaric are common constituents of fruits and vegetables including cocoa, tea, wines and whole grains (124). 3,4-dicaffeoylquinic acid and 3,5-dicaffeoylquinic acid is found in *Lonicera japonica*, a widely consumed plant (151). Globe artichoke (*Cynara cardunculus L*) also contain other derivatives of dicaffeoylquinic acid (152).

#### Tannin

Tannins are classified as condensed tannins (proanthocyanidin) and hydrolysable tannins (124). Hydrolysable tannins are further classified as gallotannins or ellagitannins based on their monomeric constituent and hydrolytic products, with gallotannins and ellagitannins possessing gallic acid and ellagic acid monomeric units, respectively (153). Tannins are common constituents of cocoa, nuts (including almonds, walnuts, and hezelnuts) and fruits (such as apples, grapes, pomegranates, plums and edible berries) (153).

#### Others Phenolic Plant Extracts

Evidence from this review also indicated that extracts from *A. melanocarpa*, blackcurrant berries, Japanese plum, *G. thunbergii*, Pomegranate fruits, *Hamamelis virginiana, Canarium album*, Brown *Alga Ecklonia*, tea and guava leaves extract suppressed respiratory viruses *in vitro*. Blackcurrant berries are edible fruits that are rich in vitamins, antioxidants and polyphenols such as flavonols, anthocyanins, and phenolic acids (154). *A. melanocarpa* (i.e., Black chokeberry) is a rich source of different polyphenols and natural antioxidants including quercetin, epicatechin, anthocyanins, hydroxycinnamic acid, and 3-caffeoylquinic (46, 155, 156). It is used in the production of wine, juices and as food grade colors (156). Japanese plum is a common type of anthocyanin–rich, red, and dark red-fleshed plums (157). *G. thunbergii* is an edible plant used for herbal preparations and likely a food additive by virtue of being listed in Korean Food Standards Codex (52). *G. thunbergii* is rich in geraniin, a type of ellagitannin which is active against different viruses like HIV-1, HSV, and IAV (52). Pomegranate fruits (*Punica granatum*) are functional food rich in different polyphenols and tannins including anthocyanins and hydrolysable tannins like isocorilagin (158). The antiviral activities of *Hamamelis virginiana* extract were associated with its tannic acid contents such as gallic acid, pentagalloylglucose, tannic acid, and EGCG (41), commonly found in many edible plants. The isocorilagin-containing edible drupe fruit of *Canarium album* (Chinese olive tree), is consumed as a digestive aid and for other medicinal values (53, 159). The antiviral activities of isocorilagin, an hydrolysable tannins, against IAV has been reported (53). Isocorilagin is also present in pomegranate fruits (158). Brown *Alga Ecklonia* is an edible algae that is widely consumed in Korea and Japan for its nutritional and health benefit (160). It is rich in polyphenols such as phlorotannins which was demonstrated to suppress influenza virus activities (40). Tea is a commonly consumed beverage drink, obtained from *Camellia sinensis* and rich in polyphenols including catechin, gallic acid, theaflavins (such as derivatives of EGCG), amino acids and alkaloids such as caffeine and theobromine (161). They are processed into black, green or oblong tea with black and green tea accounting for 78 and 20% of worldwide consumption, respectively (161). Guava leaves tea is a functional drink that is highly rich in several polyphenols including catechin, ellagic acid, and quercetin (162, 163).

#### Resveratrol, Curcumin, Germacrone

The evidence presented in this review indicates that resveratrol, curcumin and germacrone inhibited respiratory viruses by interfering with different stages of its pathological pathways. Resveratrol, a type of stilbenes polyphenol, is abundant in grapes, wine, peanuts, and peanut products (124, 164). Curcumin is a yellow colored, non-polar polyphenol abundant in tumeric (*Curcuma longa*) (124). Germacrone is a common constituent of different species of Ginger (*Zingiber spp*.) and tumeric (*Curcuma spp*.) which are commonly used as food spices and flavoring agents (165, 166).

#### Polysaccharides and Lectins

Lectins are heat and acid resistant glycoproteins that binds to specific carbohydrates moieties and are constituents of many dietary grains and legumes (167, 168). There are varieties of lectins, each having different specificity for different types of carbohydrates. Concanavalin A obtained from Jack-beans binds specifically to mannose and glucose moiety (169), peanut agglutinin, from peanut seed (*Arachis hypogaea*), binds to terminal galactose-B1,3-N-acetylgalactosamine (167), *Lens culinaris* agglutinin, from lentils, binds to fucosylated glycans (73). Jack-beans, peanut seed and lentils are consumed worldwide. Lentils are particularly rich in polyphenol (170). Lectins have been reported to have antiviral (73, 171), anti-cancers (169), antibacterial, and antifungal (171) activities. Some lectins such as concanavalin A*, Lens culinaris* agglutinin, and *Pisum sativum* agglutinin are able to interact with viral envelope and sialic acid residues of glycoproteins (171). Many lectins can escape digestion and enter enterocytes or systemic circulations in intact active forms to elicit different biologic effects (169, 172, 173). EBN is consumed amongst the Chinese for its numerous health benefits such as its immune enhancing effects (57). The antiviral effects of EBN against respiratory viruses has been attributed to its sialylglycoprotein lectin contents which has an exposed O-acetylated sialic acid moiety that can potentially interact with many viruses (57). The antiviral effect of EBN extract was enhanced on digestion with pancreatin F which generated smaller 10–25 kDa sialylglycoproteins (57). Lectin-like compounds with antiviral properties have also been isolated from Japanese plums (67). Sulphated polysaccharides from *Aphanothece sacrum* have been shown to possess activity against influenza viruses (61). *Aphanothece sacrum* is an edible blue-green algae (or cyanobacteria) that has been consumed as food over many years in Japan (174).

#### Vit D

Active forms of Vit D (1,25-dihydroxyvitamin D3, calcitriol) can modulate the expressions and activities of antiviral genes such as IFN, cathelicidin, β-defensins and ISGs (49) resulting in an enhanced virus clearance and suppressed virus–induced pathologies. Active Vit D are produced locally within different tissues from circulating 25-hydroxyvitamin D (25(OH)D3, calcidiol) through activities of 25-hydroxyvitamin D-1 alpha hydroxylase enzymes, resident in different organs such as liver, kidney, airway epitheliums etc. (72, 175). D2 and D3, obtained from the dietary sources [such as plant oil, UVB irradiated yeast cells, fish oil, mushrooms etc. (176)] or from the skin (through a UVB radiation dependent process), are converted to circulating 25(OH) Vit D through the activities of D-25-hydroxylase (175, 177).

#### Glycosides

Plant glycosides such as glycyrrhizin, acteoside, geniposide, and iridoid glycosides were observed to possess antiviral effects against respiratory viruses. Glycyrrhizin, a glycosylated saponin (178), is the major bioactive constituent of *G. uralensis* extract (20). *G. uralensis*, also known as licorice, is used in herbal preparation and also consumed as food supplement in many parts of the world (20). Extracts from *G. uralensis* roots also contains flavonoids, chalcones and other agents with estrogenic activities (179). Metabolism of Glycyrrhizin by gut microorganisms generated 18β-GA as its primary metabolite (20). 18β-GA has been shown to inhibit RSV and induced inflammatory responses (20). Acteoside is a phenylethanoid glycoside with potent anti-inflammatory properties. It is the bioactive constituent of cistanche tea (from *Cistanche spp*), which is commonly consumed as herbal and functional drink in many Asian countries (180). Acteoside is also present in Kuding tea (prepared from leaves of *Ilex kudingcha*), a commonly consumed polyphenol-rich functional drink in Asian countries (181). Geniposide and other iridoid glycosides isolated from *Fructus Gardenia* and *Gardenia jasminoides* J. Ellis fruit have been demonstrated to possess antiviral properties against influenza viruses (50). Iridoid are monoterpenoids with a six membered ring structure and often associated with carbohydrates (182). Iridoid are also present in honeysuckle berry, guelder berry, *Morinda citrifolia, Cornus officinalis*, and *Olea europaea* which are edible dietary components (183–186). Iridoids have been reported to have anti-inflammatory, antibacterial, and antiglycation properties (183, 185).

#### Adlay Tea, Houttuynia Cordata, Rumex Acetosa, Portulaca Oleracea, and Adansonia Digitata Extracts

This review showed that extracts from adlay tea, *H. cordata, Rumex acetosa, P. oleracea*, and *A. digitata* inhibited respiratory virus *in vitro*. Adlay tea is consumed in parts of Asia and made from ingredients consisting of adlay seeds (*Coix lacryma-jobi*), cassia (*Cassia obtusifolia L*.), soybeans (*Glycine max*), and barley (*Hordeum vulgare var. nudum*) seeds (16). The antiviral effects of adlay tea have been associated with its non-polyphenolic contents (16). *H. cordata Thunb* is used in herbal preparations and consumed as edible vegetable in some parts of Asia (187, 188). It is rich in volatile oil, water soluble polysaccharide and flavonoid, and reported to exerts anti-inflammatory, antimutagenic, and antiviral activities (187, 188). *H cordata* have been reported to enhance the secretion of interleukin and increase the proliferation of CD4+ and CD8+ cells in response to SARS coronavirus infection (187). Some of the antiviral bioactivities of *H. cordata* have been attributed to its quercetin content (21, 187). *Rumex acetosa L* is a wild vegetable, rich in important nutrients but contains a high level of oxalic acid (189). The succulent leaves are consumed as vegetable and as medicinal plants (189). Purslane (*P. oleracea*) is an edible leafy plant with a high nutritive value as well as contains high levels of the antinutrient, oxalic acid (190). The leaves, seeds and fruit of *A. digitata* are consumed in many African countries as food and also used in traditional medicine for their antioxidant and anti-inflammatory properties (191).

#### Summary and Recommendations

This review highlighted the potential benefits of different PDFGS agents against viruses that can infect human respiratory tracts. Cumulatively, the evidence presented in this review shows that some PDFGS posses both *in vitro* and *in vivo* activities against different respiratory viruses. However, these evidence needs to be interpreted with cautious since it has not been demonstrated in human models of respiratory viral infection. As such, further clinical studies are required to ascertain their anti-viral potencies in human subjects. In the absence of more conclusive evidence from human studies, we recommend the following nutrition amongst populations that are highly prone to respiratory virus infections. These recommendations are based largely on the *in vitro* data presented in this review as well as on the merits of other nutritive health benefits inherent in the PDFGS.

We recommend an increased intake of many edible fruits and tea due to their abundant flavonoids, phenolic acids, and tannin contents.

We recommend an increased intake of legumes and other lectin-rich foods due to their abundant lectin contents. Increased consumption of fruits, and legumes was recently observed to lower the mortality due to COVID-19 infection (35, 192).

We recommend an increased culinary usage of spice herbs such as tumeric, ginger, garlic due to their curcumin, and germacrone content.

We recommend the consumption of plant oils, fatty fish, beef liver, egg yolks, and other sources of Vit D precursors as well as a decent exposure to sunlight or other UVB radiations may boost overall immunity against respiratory viruses and other infectious diseases (177). The beneficial effect of Vit D is further supported by recent studies that associated the severity of the COVID-19 infection with Vit D deficiency (193–195).

Decent consumption of herbal teas such as licorice tea, cistanche tea, kuding tea, EBN, adlay tea due to their antiviral components. Glycyrrhizin from licorice tea was recently proposed as a potential therapeutic agent against COVID-19 as a result of its anti-inflammatory and angiotensin-converting enzyme 2 (ACE2) suppressing capabilities (196).

#### Limitation of the Study

This systematic review is limited by all the various limitations of the individual studies used for the review. The review is also limited by the inherent deficiencies of systematic reviews one of which includes a reliance on only available literature. For instance, majority of the available evidences were derived from *in vitro* studies as well as from IAV models of respiratory virus infections. This suggests that findings from this review may not be generalized for all kinds of respiratory viruses or for animal or clinical manifestations of IAV infections. This review is also limited by possible omissions of some relevant studies due to the choices of keywords used as well as the choice of databases used for literature search. The potential presence of publication bias in the information used for this review was not tested and cannot be ruled out.

## Conclusions

This systematic review presented evidence that showed the antiviral potentials of PDFGS against cells and animal models of respiratory virus infections. Respiratory viruses susceptible to some of the PDFGS highlighted in this review includes IAV (H1N1, H2N1, H3N2, H9N2, and H5N1 subtypes), IBV, AdV, RSV (A2, B, and long strain), mouse hepatitis virus, hPIV (type 2 and type 3), RV1B and HCoV-NL63. PDFGS including flavonoids (such as quercetin and isorhamnetin), phenolic acids (such as caffeic), tannins (such as proanthocyanidin and ellagitannins), lectins (such as concanavalin A, peanut agglutinin, and Lens culinaris agglutinin), glycosides (such as glycyrrhizin, acteoside, geniposide, and iridoid glycosides), resveratrol, curcumin, germacrone, Vit D, and plant extracts (such as adlay tea, *H. cordata, Rumex acetosa, P. oleracea*, EBN, and *A. digitata*) were shown to be active against some categories of these respiratory viruses. These PDFGS inhibited different stages of respiratory virus's pathogenic life cycle including virus cell entry, replication, protein synthesis and release functions. The PDFGS also enhanced host antiviral immune response as well as suppress virus-induced cellular damages to host tissues. Low bioavailability of these PDFGS in human subjects as well as the *in vitro* nature of the evidence presented in this review potentially limits the translation of this evidence to clinical practices. This notwithstanding, we are of the opinion that a nutrition that is rich in a number of the PDFGS highlighted in this study, would act at multiple points in respiratory virus's pathogenic life cycle, to create a cumulative antiviral effect which could limit the infectivity, proliferation or virus-induced cellular damage of respiratory viruses. The validity of this claim is a subject for future animal and clinical studies.

## Data Availability Statement

The original contributions presented in the study are included in the article/supplementary material, further inquiries can be directed to the corresponding author/s.

## Author Contributions

FU participated in literature search, data extraction, wrote the results and discussion section, conceptualized and designed the Figures 5–8, and collated the entire work. BE-E participated in literature search, data extraction, and wrote the introduction section. KP-I and JZ reviewed manuscript for clarity. OO supervised, conceptualized the project, and reviewed manuscript for clarity. All the authors read and approved the final manuscript.

## Conflict of Interest

The authors declare that the research was conducted in the absence of any commercial or financial relationships that could be construed as a potential conflict of interest.

## Abbreviations

18β-GA: 18β-glycyrrhetinic acid
*A. Annua*: *Artemisia annua*
*A. digitata*: *Adansonia digitata*
*A. melanocarpa*: *Aronia melanocarpa*
ACE: Angiotensin-converting enzyme
ACE2: Angiotensin-converting enzyme 2
AdV: Adenovirus
AKT: Protein kinase B
AP-1: Activator protein-1
CC50: 50% cytotoxic concentration
Cmax: Peak plasma level
COPD: Chronic obstructive pulmonary disease
DMO-CAP: 6-demethoxy-4′-O-methylcapillarisin
EBN: edible bird nest (EBN)
EGCG: Epigallocatechin gallate
ERK: Extracellular signal-regulated kinase
*G. thunbergii*: *Geranii thunbergii*
*G. uralensis*: *Glycyrrhiza uralensis*
GSP: Grape seed proanthocyanidin
*H. cordata*: *Houttuynia cordata*
HA: hemagglutinin
HA2: Fusion peptide
HCoV-NL63: Human coronavirus NL63
hPIV: Human parainfluenza virus
hPIV-2: Human parainfluenza virus type 2
hPIV-2-F: hPIV-2 fusion protein
hPIV-3: Human parainfluenza virus type 3
HS: *Hibiscus sabdariffa*
i.p: Intraperitoneal
IAV: Influenza A virus
IAV-M: IAV matrix protein
IAV-P: IAV polymerase
IBV: Influenza B virus
IC50: Half maximal inhibitory concentration
IFITM: IFN-induced transmembrane
IFN: Interferons
IFN-α: Interferon α
IFN-β: Interferon β
IFN-γ: Interferon γ
IKK: IkB kinase
IL-18: Interleukin 18
IL-1β: Interleukin 1 β
IL-6: Interleukin 6
iNOS: Inducible nitric oxide synthase
IRF: Interferon regulatory factor
IRF3: Interferon regulatory factor 3
ISGs: Interferon-stimulated genes
JAK1: Janus kinase 1
LLC-MK2: Rhesus Monkey Kidney Epithelial Cells
M1: Matrix protein 1
M2: matrix protein 2
MAPK: Mitogen-activated protein kinase
MDA-5: Melanoma differentiation-associated protein 5
MHV: Mouse hepatitis virus
MMP: Pulmonary matrix metalloproteinase
mRNA: Messenger ribonucleic acid
MxA: Myxovirus Resistance Gene A
MxB: Myxovirus Resistance Gene B
NA: Neuraminidase
NF-κB: Nuclear factor-κB
NLRP3: NLR family pyrin domain containing 3
NP: nucleoprotein
Nrf2: Nuclear factor erythroid 2-related factor 2
NS1: Non-structural protein 1
NS2: Non-structural protein 2
OAS: 2′-5′ oligoadenylate synthetase
*P. oleracea*: *Portulaca oleracea*
PA: Polymerase acidic protein
PABPII: Polyadenine binding protein II
PAMP: Pathogenic associated molecular patterns
PB1: Polymerase basic protein 1
PB1-F2: Polymerase basic protein 1–F2
PB2: Polymerase basic protein 2
PDFGS: Plant-derived food grade substances
PI3K: Phosphatidylinositol-3-kinase
PIVs: Parainfluenza viruses
PKR: Protein kinase R
PPR: Pattern recognition receptors
*R. acetosa*: *Rumex acetosa*
RANTES: Regulated on activation normal T cell expressed and secreted
RIG-1: Retinoic acid-inducible gene I
RNA: Ribonucleic acid
RNase L: Ribonuclease L
RNP: Ribonucleoprotein
ROS: Reactive oxygen species
rPR8-GFP: Recombinant H1N1/PR8 expressing green fluorescent protein
RSV: Respiratory syncytial virus
RSV-F: RSV fusion protein
RSV-G: Glycoprotein
RSV-L: RSV RNA-dependent RNA polymerase
RSV-M2-1: RSV matrix M2-1
RSV-M2-2: RSV matrix M2-2
RSV-N: RSV nucleoprotein
RSV-NS2: RSV non-structural protein 2
RSV-P: RSV phophoprotein
RSV-RNP: RSV ribonucleoprotein
RSV-SH: RSV hydrophobic protein
RV: Rhinovirus
RV1A: Rhinovirus 1A
RV1B: Rhinovirus 1B
SARS-CoV-2: Severe acute respiratory syndrome coronavirus 2
ssRNA: Single-stranded RNA
STAT1/2: Signal transducer and activator of transcription 1/2
T-bet: T-box expressed in T cells
TCCM: Tissue culture cell monolayers
TLR: Toll-like receptor
TLR4: Toll-like receptor 4
TNF-α: Tumor necrosis factor-α
TRAF6: Tumor necrosis factor receptor (TNFR)-associated factor 6
TRIF: TIR-domain-containing adapter-inducing interferon-β
TRIM25: Tripartite motif containing 25
TYK2: Non-receptor tyrosine-protein kinase
*U. lactuca*: *Ulva lactuca*
Vit D: vitamin D
vRNP: Viral ribonucleoprotein
wnt3a: Wnt family member 3a
*Z. officinale*: *Zingiber officinale*.
